# Electrophysiological Signatures of Perceiving Alternated Tone in Mandarin Chinese: Mismatch Negativity to Underlying Tone Conflict

**DOI:** 10.3389/fpsyg.2021.735593

**Published:** 2021-09-27

**Authors:** Yuyu Zeng, Robert Fiorentino, Jie Zhang

**Affiliations:** ^1^Phonetics and Psycholinguistics Laboratory, Department of Linguistics, University of Kansas, Lawrence, KS, United States; ^2^Neurolinguistics and Language Processing Laboratory, Department of Linguistics, University of Kansas, Lawrence, KS, United States

**Keywords:** phonological alternation, underlying representation, surface representation, mismatch negativity, Mandarin 3rd tone sandhi, spoken word recognition, lexical tone, event-related potentials

## Abstract

Although phonological alternation is prevalent in languages, the process of perceiving phonologically alternated sounds is poorly understood, especially at the neurolinguistic level. We examined the process of perceiving Mandarin 3rd tone sandhi (T3 + T3 → T2 + T3) with a mismatch negativity (MMN) experiment. Our design has two independent variables (whether the deviant undergoes tone sandhi; whether the standard and the deviant have matched underlying tone). These two independent variables modulated ERP responses in both the first and the second syllables. Notably, despite the apparent segmental conflict between the standard and the deviant in all conditions, MMN is only observed when neither the standard nor the deviant undergoes tone sandhi, suggesting that discovering the underlying representation of an alternated sound could interfere with the generation of MMN. A tentative model with three hypothesized underlying processing mechanisms is proposed to explain the observed latency and amplitude differences across conditions. The results are also discussed in light of the potential electrophysiological signatures involved in the process of perceiving alternated sounds.

## Introduction

### When Two Underlying Representations Are Mapped to One Surface Representation

Most theories of phonology differentiate underlying representation (UR, henceforth) and surface representation (SR, hereafter; see Cole and Hualde, 2011, for a recent in-depth discussion of this topic). Understanding how UR and SR are related is one of the driving forces behind phonological studies and speech science research in general. Traditional rule-based phonology expresses this UR-SR relationship with rules. For example, there are four contrastive lexical tones (termed T1, T2, T3, and T4, henceforth) in Mandarin Chinese (Mandarin, hereafter). The canonical realization of an isolated T3 syllable has a low fundamental frequency (F0) (Moore and Jongman, 1997; Prom-on et al., 2009; Kuang, 2017). When two underlyingly T3 syllables occur consecutively, the first T3 syllable's SR has a rising F0 contour (Xu, 1994; Yuan and Chen, 2014), perceptually indistinguishable from an underlying T2's canonical SR in the same position (Xu, 1994; Chen et al., 2015)^1^. This 3rd tone sandhi in Mandarin can be formalized with a rule:


$$\begin{array}{l} T3+T3\;\to T2+T3 \end{array}$$

In Mandarin, the SR of a T3 syllable alternates according to its phonological context, leading to one of its SRs being perceptually indistinguishable from a T2 syllable's canonical SR. In this example, two distinct URs (T2 and T3) appear to be mapped onto the same SR (a rising F0 contour) in a potentially sandhi-triggering environment (before another T3). This type of phonological alternation^2^, termed neutralization, obscures the distinct contrast between two URs, creating a temporary SR overlap. For native Mandarin listeners, upon hearing an upward-moving F0 contour, they need to infer the correct UR by taking its context (the next syllable's UR) into account.

The present study investigates how listeners uncover the correct UR during speech perception when faced with a potentially ambiguous SR stemming from phonological alternation, and specifically, the corresponding neurophysiological underpinnings of this SR-UR mapping process. The 3rd tone sandhi in Mandarin introduced above will be our test case: upon hearing a rising F0 contour, how do listeners uncover the correct underlying tone?

### Mismatch Negativity as a Tool to Examine Access to the UR

The concept of the phoneme is closely related to the concept of the UR. Two distinct URs must involve two contrastive phonemes in a language (see Dresher, 2011, for a detailed discussion). Besides behavioral tasks (e.g., Jaeger, 1980), electrophysiological tools such as EEG (electroencephalography) and MEG (magnetoencephalography) have also been used to demonstrate the psychological reality of the phoneme (Näätänen et al., 1997; Phillips et al., 2000).

The mismatch negativity (MMN) and its MEG counterpart MMNm are a primary area of interest in electrophysiological studies on language processing (see Shtyrov and Pulvermüller, 2007, for a review). MMN(m) arises from electrical activity in the brain when an incoming stimulus is presented in an oddball paradigm: one oddball stimulus (the “deviant”) is placed after multiple homogenous stimuli (the “standards”). For example, the voiced alveolar stop [d] and the voiceless alveolar stop [t] are contrastive (i.e., differentiating meaning) in Russian but are allophonic (i.e., not differentiating meaning) in Korean. In an MEG study presenting [t] among repetitions of [d], Kazanina et al. (2006) found MMNm among Russian native speakers; this MMNm response was absent among native Korean speakers. The authors concluded that the language-specific phoneme inventories modulated the presence/absence of MMNm: the psychological reality of language-specific phoneme representations is corroborated by the presence or absence of MMNm, depending on whether two sounds are contrastive or allophonic in a tested language.

For speech sounds, MMN has been shown to index contrasts in vowel (Näätänen et al., 1997), consonant (Phillips et al., 2000; Kazanina et al., 2006), lexical tone (Kaan et al., 2007; Yu et al., 2017), duration (Colin et al., 2009), and stress (Honbolygó and Csépe, 2013; Honbolygó et al., 2017). However, the majority of these studies focused on phonemes without phonological alternation. In these studies, there is usually a simple one-to-one SR-UR mapping, so little is known about how the SR of an alternated sound is mapped to its UR during speech perception, not to mention the corresponding electrophysiological signatures associated with this SR-UR mapping process.

As concerns Mandarin 3rd tone sandhi, which is the focus of the current study, an emerging literature has begun to examine the representation of T3, primarily utilizing monosyllabic stimuli as opposed to disyllabic, sandhi-inducing contexts. In Li and Chen (2015) and Politzer-Ahles et al. (2016), native Mandarin listeners heard isolated T2 and T3 in their respective canonical forms. Both studies found that when T2 is the standard and T3 the deviant, the elicited MMN has a higher amplitude than the reverse (i.e., T3 as the standard, and T2 as the deviant). Such MMN asymmetry when the standards and deviants are swapped was not observed between T1 and T3 in Li and Chen (2015). Li and Chen (2015) interpreted their results as support for the storage of T3 tonal variants in the mental lexicon. In contrast, Politzer-Ahles et al. (2016) found an MMN asymmetry when other Mandarin tone pairs were contrasted (e.g., T3 and T4), suggesting perceptual contributions to MMN asymmetries. However, when non-native listeners were tested, the predicted T2/T3 MMN asymmetry was absent. Politzer-Ahles et al. (2016) interpreted this result as stemming from an underspecified T3: it is hypothesized that when an underspecified sound serves as the standard, its lack of specification in the native mental lexicon (hence the term “underspecification”) makes a conflict between the standard and the deviant less severe, leading to a smaller MMN (Avery and Rice, 1989; Lahiri and Reetz, 2010; Alexandrov et al., 2011). Chang et al. (2019) contrasted Mandarin T1 and T3, T2 and T3, in an MMN experiment; for each pair, standards/deviants in both directions were tested, and the data were analyzed by collapsing across standard/deviant direction (e.g., T1 standard/T3 deviant, and T3 standard/T1 deviant). They found that the latter pair had later latency and weaker amplitude than the former pair. Combined with their results from testing Taiwanese tone sandhi^3^ pairs, which showed that ERP responses were modulated by listeners' productivity of the Taiwanese tone sandhi, they attributed their Mandarin results to the Mandarin 3rd tone sandhi rule (a phonological substitution rule). Note that all stimuli in these three studies are monosyllabic, so T3 and T2 are acoustically distinct, and the sandhi-triggering context is not met. Consequently, their results do not directly inform us of the process of mapping the SR of an alternated sound to its UR. More recently, Chien et al. (2020) examined T3 in context. Their standards were disyllabic in all conditions, while the deviant remained the same: a monosyllabic T2. This is the only study we are aware of that tested Mandarin 3rd tone sandhi with disyllabic stimuli. In their Tone 2 Condition, the standards were underlyingly T2 + T4; in their Tone 3 Condition, the standards were underlyingly T3 + T4; in their Sandhi Condition, the standards were underlyingly T3 + T3; in their Mixed Condition, the standards were either T3 + T4 or T3 + T3 underlyingly. Critically, the Tone 2 Condition (standard: T2 + T4) and the Sandhi Condition (standard: T3 + T3 → T2 + T3) had the same rising F0 in the first syllable (S1, henceforth), which resembles the SR of the T2 deviant.

Interestingly, only the Tone 2 Condition (standard: T2 + T4) and the Tone 3 Condition (standard: T3 + T4) yielded MMN in S1; MMN was absent in S1 of the Sandhi Condition (standard: T3 + T3 → T2 + T3) or the Mixed Condition (standard: T3 + T4 or T3 + T3 → T2 + T3). Chien et al. (2020) interpreted their results as suggesting either an underspecified representation (cf. Archangeli, 1988; Avery and Rice, 1989; Mohanan, 1991; Lahiri and Reetz, 2010) or an underlying T3 representation of the 3rd tone sandhi form: if the 3rd tone sandhi form is stored as T2 in the mental lexicon, the Tone 2 Condition (standard: T2 + T4) and the Sandhi Condition (standard: T3 + T3 → T2 + T3) should have yielded identical results.

Chien et al. (2020) demonstrated the influence of the UR via the dissociation between the Tone 2 Condition (standard: T2 + T4) and the Sandhi Condition (standard: T3 + T3 → T2 + T3), suggesting that even for a phonologically alternated sound that created a potentially ambiguous SR, such as the Mandarin 3rd tone sandhi, UR could be accessed in a passive-listening MMN experiment. This is also consistent with studies showing MMN's sensitivity to morphological processes such as inflection, derivation, and compounding (see Leminen et al., 2019, for an extensive review). Therefore, we hypothesize that when information regarding the correct UR is available, the brain can access the UR of alternated sounds in a passive-listening MMN experiment. In the Mandarin 3rd tone sandhi example, this should occur when the targeted T3 sandhi form is placed in its appropriate context (i.e., before another T3).

### The Present Study

Despite the cross-linguistic prevalence of phonological alternation, relatively little is known regarding how the SR of an alternated sound is mapped to its UR during speech perception and the corresponding electrophysiological signatures. The present study aims to advance the literature on these questions using the Mandarin 3rd tone sandhi as a test case.

The study uses the well-known MMN method, as previous research was lacking to provide a definitive answer regarding the best experimental paradigm to test the perception of alternated sounds. Table 1 shows our experimental conditions. Our study adopts a 2 ^*^ 2 design according to: (1) whether the deviant is underlying T2 + T3 or T3 + T3 (Non-sandhi deviant vs. Sandhi deviant); (2) whether the standard and the deviant conflict at the UR tone level (UR-match vs. UR-mismatch). These two independent variables will be referred to as the *Deviant Sandhi Status* and the *UR Relation* hereafter.

**Table 1:** A summary of experimental conditions.

**Condition label**	**Standard**	**Deviant**	**Segmental conflict?**	**UR tone conflict?**	**Direct SR-UR mapping?**
UR-match and Non-sandhi deviant (#1)	T2 + T3	T2 + T3	Yes	No	Yes
UR-match and Sandhi deviant (#2)	T3 + T3	T3 + T3	Yes	No	No
UR-mismatch and Non-sandhi deviant (#3)	T3 + T3	T2 + T3	Yes	Yes	Yes
UR-mismatch and Sandhi deviant (#4)	T2 + T3	T3 + T3	Yes	Yes	No

*The first column introduces the label for each condition and its respective condition number (in brackets). The next two columns show the underlying tone for the standards and the deviants. The next column, “Segmental conflict?” highlights the presence of segmental conflict in all conditions. The column “UR tone conflict?” indicates whether there is UR tone conflict in a condition. The last column, “Direct SR-UR mapping?” indicates whether the deviant is T2 + T3 (direct SR-UR mapping) or T3 + T3 (indirect SR-UR mapping)*.

The two levels of *Deviant Sandhi Status* (Non-sandhi deviant vs. Sandhi deviant) differ according to whether the S1 of the deviant is underlyingly T2 or T3. Our working hypothesis assumes T2 as the default upon hearing a rising F0, which is the canonical realization of T2. Under this working hypothesis, when a deviant is underlyingly T2 + T3, the brain engages in a direct SR-UR mapping; in contrast, when a deviant is underlying T3 + T3, the brain needs to override its default (T2) to uncover the correct UR (T3) (see the column “Direct SR-UR mapping” of Table 1). This working hypothesis also echoes the findings of Zhang et al. (2015), an EEG production study in Mandarin. Zhang et al. (2015) showed that when compared to producing a T2 + T3 word, producing a T3 + T3 word requires more effortful phonological encoding.

Segmental conflict is present in all conditions. This inclusion of segmental conflict is inevitable: we need a stimulus to have a unique underlying tone in its S1; otherwise, the stimuli will be ambiguous homophones. For example, a surface [bai^2^ mi^3^] in Mandarin can be either /bai^2^ mi^3^/ (*white rice*) or /bai^3^ mi^3^/ (*one hundred meters*) underlyingly^4^. It is impossible to design an MMN experiment with such homophones, as the standard and the deviant would be acoustically near-identical. To ensure that the standard and the deviant have a uniquely identifiable UR, we introduced segmental conflict. Due to the presence of segmental conflict, ERP responses time-locked to the onset of the deviants should yield MMN. Put differently, due to the presence of a segmental conflict in all conditions, we predict a segmental MMN in all conditions, occurring ~150–250 ms from the deviant's onset (Näätänen et al., 2007).

In Table 1, only the UR-match and Non-sandhi deviant Condition (#1) has a direct SR-UR mapping for both the standards and the deviants; in the other three conditions, the brain needs to perform extra processing operation(s) to arrive at the correct UR for at least one of the stimulus categories (standard and deviant). Such extra processing operation(s) may interfere with the generation of the predicted segmental MMN, thus modulating MMN in conditions other than the UR-match and Non-sandhi deviant Condition (#1). In the extreme, the predicted segmental MMN would be absent: if such a scenario (the absence of segmental MMN) occurs, it would serve as indirect evidence for the SR-UR mapping process [see, e.g., the results of Chien et al. (2020) summarized in section Mismatch Negativity as a Tool to Examine access to the UR].

Our two independent variables are also related: the *Deviant Sandhi Status* needs to be correctly inferred before a final decision regarding the *UR Relation* can be made. Such a dependency entails that a *Deviant Sandhi Status* effect should occur before a *UR Relation* effect. This is not to say that the SR-UR mapping process has to be completed before a decision regarding the *UR Relation* can be attempted. Numerous studies have shown that speech perception is incremental (Fernald et al., 2001; Magnuson et al., 2003; McQueen and Viebahn, 2007; Rayner and Clifton, 2009, to cite only a few). We also assume incrementality/cascading between perceptual processing stages: although the processing for the *Deviant Sandhi Status* should precede the processing for the *UR Relation*, the two processes could overlap temporally.

ERP responses to the entire disyllabic deviants will also be inspected. In the case of the Mandarin 3rd tone sandhi, the second syllable (S2, henceforth) informs listeners of the correct UR of S1, so the effects of the SR-UR mapping (for uncovering the *Deviant Sandhi Status*) and *UR Relation* could also be present in S2. A careful examination of the ERP waveforms as processing unfolds, especially in the S1-S2 transitional region, is necessary to ascertain whether the observed S2 effects are genuinely attributable to S2, instead of spill-over artifacts from S1 (see Steinhauer and Drury, 2012, for an in-depth discussion on the possibility of mistaking spill-over effects for target responses).

For the moment, the timing and the topographic distribution of the SR-UR mapping process (the effect of the independent variable *Deviant Sandhi Status*) and the decision regarding the *UR Relation* are unknown. Thus, our study has an exploratory side: the effects of *Deviant Sandhi Status* and *UR Relation* will be revealed by analysis via comparison of conditions. If we observe an effect of our independent variables, it would serve as indirect evidence for the SR-UR mapping process. Such a finding (our independent variables modulating ERP responses) will also show that the brain can access the UR in a passive-listening MMN experiment.

In sum, our study investigates the process of mapping the SR of the alternated T3 in Mandarin to its UR using the passive-listening MMN procedure. Our study is simultaneously confirmatory and exploratory. On the confirmatory side, we predict segmental MMNs in the S1 of all conditions, while interference of these S1 segmental MMNs is also possible in conditions where extra processing effort is needed to uncover the correct UR for sandhi words. Such MMN interference, if it occurs, would serve as indirect evidence for the SR-UR mapping operations. On the exploratory side, because the existing literature has not yet established which ERP response(s) could be linked to the SR-UR mapping process and UR conflict, we employed the cluster-based permutation method, which is better suited for revealing significant differences across conditions without a priori specification of regions of interest or time windows of interest (Maris and Oostenveld, 2007). We explore whether ERP responses are modulated by our independent variables (*UR Relation* and *Deviant Sandhi Status*) and their interaction. Because the emergence of a *UR Relation* effect and a *Deviant Sandhi Status* effect is contingent on a correct SR-UR mapping outcome, if we find that our independent variables modulate ERP responses, such results will also serve as indirect evidence for the SR-UR mapping process.

## Methods

### Stimuli

Four meaningful Mandarin disyllabic words/phrases were selected, summarized in Table 2^5^. All phonetic notations used in this paper are IPA (International Phonetic Alphabet). All lexical items in Table 2 have the same S2: 马, /ma^3^/, *horse*. All lexical items differ in their S1 both segmentally and tonally: two are underlyingly T2 + T3, and the other two underlyingly T3 + T3. After applying the 3rd tone sandhi, all four S1s have the same surface tone realization: a rising F0 contour, which is the canonical realization of T2. Previous studies have shown that MMN is sensitive to lexical properties such as lexical frequency and lexicality of the stimuli tested (Jacobsen et al., 2004; Shtyrov et al., 2011). Thus, although our selected lexical items have identical surface F0 realizations, it is assumed that their lexical information will guide the listeners to the correct UR, as the alternatives are highly improbable (see the last row of Table 2).

**Table 2:** Relevant information of lexical items used in the present study (the phonetic notations are IPA).

**Written form**	**河马**	**皇马**	**好马**	**海马**
Meaning	*hippo (river horse)*	*Real Madrid C.F. (royal horse)*	*good horse*	*seahorse (sea horse)*
Underlying representation (UR)	/xə^2^ ma^3^/	/xuaŋ^2^ ma^3^/	/xaʊ^3^ ma^3^/	/xai^3^ ma^3^/
Surface representation (SR)	[xə^2^ ma^3^]	[xuaŋ^2^ ma^3^]	[xaʊ^2^ ma^3^]	[xai^2^ ma^3^]
Frequency per million characters^a^	1.85	4.82	1.04	0.84
Alternative interpretation of the SR	/xə^3^ ma^3^/	/xuaŋ^3^ ma^3^/	/xaʊ^2^ ma^3^/	/xai^2^ ma^3^/
	Non-existent	Non-existent	*tycoon horse*	*kid horse*

a *Accessed on August 3rd, 2021 from the Internet corpus (http://corpus.leeds.ac.uk/frqc/internet-zh.num; corpus size: 280 million Chinese words)*.

*All four lexical items have the same second syllable (S2). The first two lexical items are underlyingly T2 + T3, and the last two lexical items are underlying T3 + T3. The last row shows potential alternative interpretations of the surface representation (SR): as illustrated by the last row, our selection of the lexical items guides the listeners to the underlying representation (UR) we intended, as the alternatives are highly improbable*.

**Figure 1:**
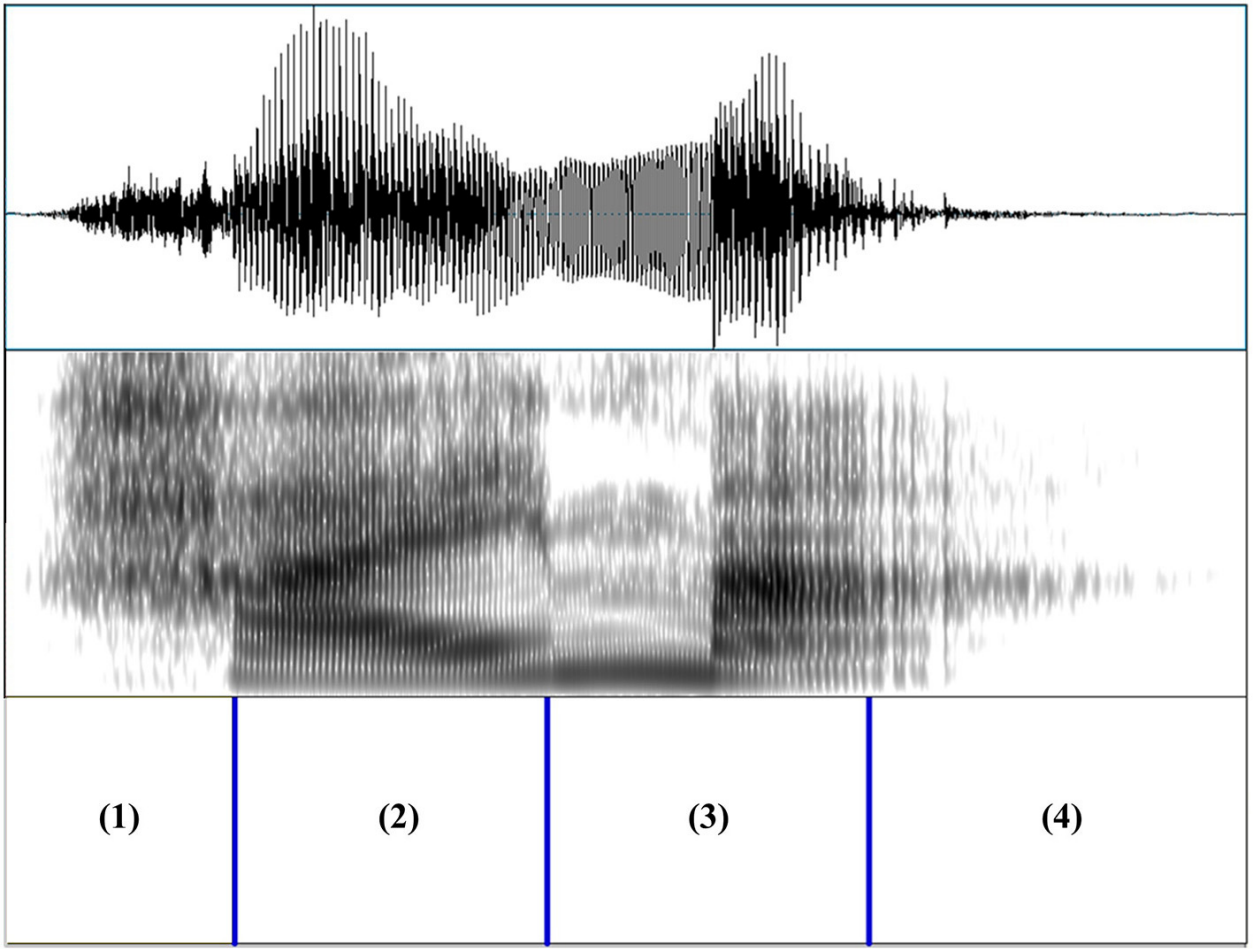
Demarcation of one base token (example: 海马, /xai^3^ ma^3^/, seahorse) before manipulation: (1) the initial fricative /x/ without vocal-fold vibration; (2) the voicing portion of the first syllable (S1); (3) the voicing portion of the second syllable (S2); (4) the end of S2 characterized by strong creaky voice, not amenable to F0 manipulation.

A female native speaker of Mandarin (aged 29) read the four lexical items in isolation in an anechoic chamber. Four tokens with similar F0 trajectories were selected as the base for manipulation for minimal modification of the original recordings. All manipulations were performed in Praat (Boersma and Weenink, 2020). The mean-energy intensity of each token was first scaled to 70 dB. Durations and mean-energy intensities of the following four portions were then measured: (1) the initial fricative /x/ without vocal-fold vibration; (2) the voicing portion of S1; (3) the voicing portion of S2; (4) the end of S2 characterized by strong creaky voice, not amenable to F0 manipulation. Figure 1 illustrates the demarcation of these four portions.

**Figure 2:**
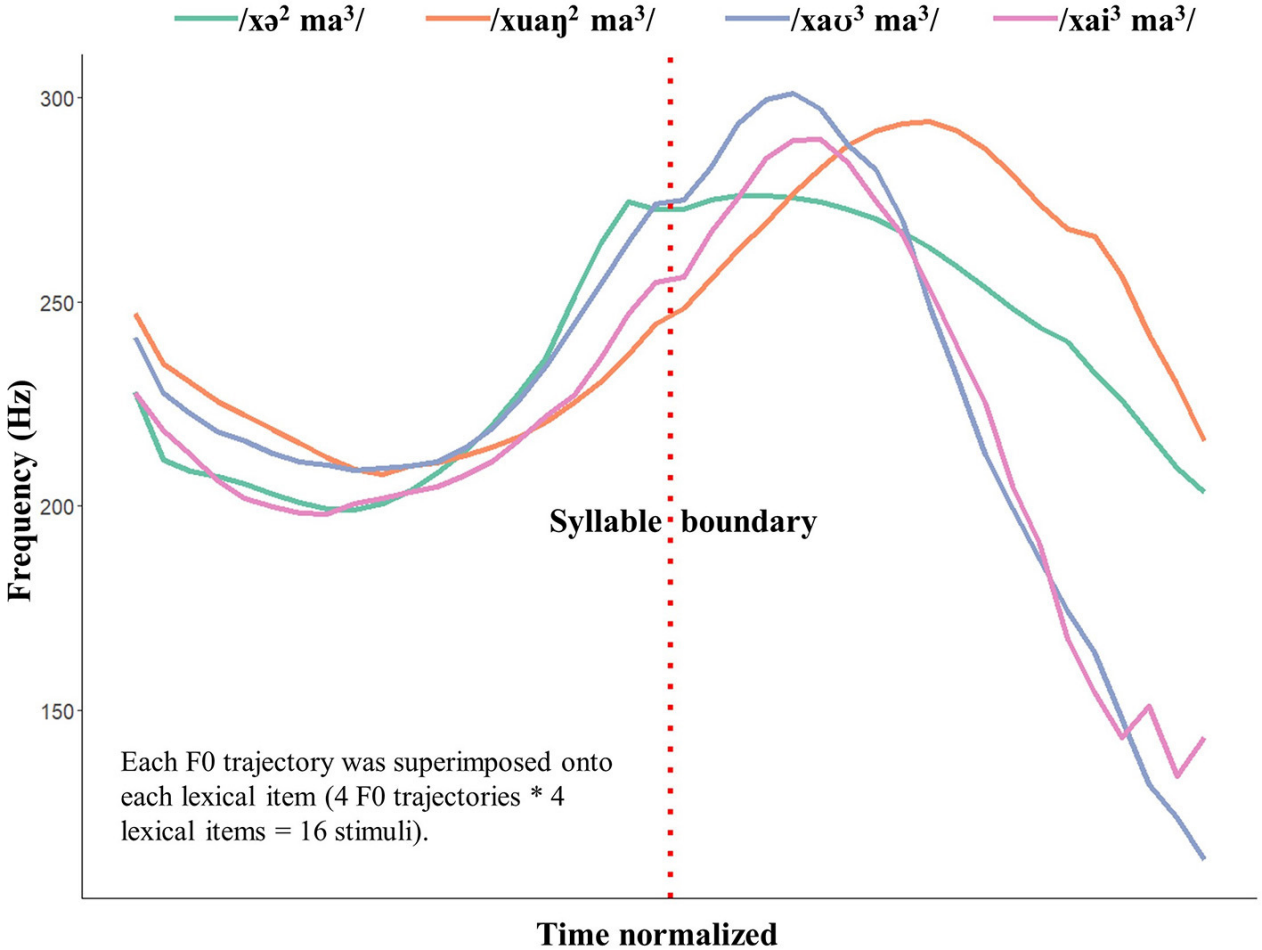
F0 trajectories of the base tokens (duration normalized). Each of the four F0 trajectories is superimposed onto each duration-normalized base token, leading to the 16 stimuli used in our study (4 lexical items * 4 F0 trajectories).

The measured duration and mean-energy intensity for each portion, together with their averages across the four tokens for that portion, are included in the Supplementary Tables 1, 2. The duration and the mean-energy intensity of each of the four portions were then adjusted to their averages. Previous studies have argued that oddball paradigms with multiple renditions of the standards and the deviants provide stronger support for processing at a linguistically meaningful level (Phillips et al., 2000; Politzer-Ahles et al., 2016; Yu et al., 2017). In line with these studies, F0 variability was introduced. Four renditions of each lexical item were created using the original F0 trajectories of the four base tokens (see Figure 2).

Portions 2 and 3 of the base tokens (Figure 1) were measured with 20 equally timed F0 points. The four duration and intensity equalized tokens were then resynthesized according to the original base tokens' F0 trajectories using the Overlap-and-Add method (Moulines and Charpentier, 1990). This way, four renditions were generated for each lexical item. For example, four renditions of the word /xə^2^ ma^3^/ (*hippo*) were created, modeling on the F0 trajectories of the original /xə^2^ ma^3^/ (*hippo*), /xuaŋ^2^ ma^3^/ (*Real Madrid C.F*), /xaʊ^3^ ma^3^/ (*good horse*), and /xai^3^ ma^3^/ (*seahorse*), respectively. Consequently, a total of 16 stimuli were generated (4 lexical items ^*^ 4 F0 trajectories). Sound files of these 16 stimuli can be found in the Supplementary Material.

Finally, the overall duration of each stimulus was slightly shortened without seriously sacrificing perceived naturalness, and the mean-energy intensity of each stimulus was scaled to 70 dB. For the final stimuli, S1 lasted 390 ms, and S2 lasted 430 ms, a total of 820 ms. A phonetically trained experimenter (also a native speaker of Mandarin) other than the creator of the stimuli verified that after the above manipulation, all stimuli sounded natural.

In short, the above manipulation procedure ensured: (1) the variability of the stimuli for evaluating linguistically relevant effects; (2) comparable acoustic profiles of the stimuli, especially for the critical F0 cues; (3) that all stimuli sounded natural.

### Participants

Thirty-one (31) healthy adult native Mandarin speakers (eight males; mean age = 23.72, SD = 4.9) volunteered for our study. Monetary compensation was provided. This study was reviewed and approved by the Human Subjects Committee at the University of Kansas (STUDY00144397). All participants provided informed consent.

A participant had to meet the following criteria before they could participate: (1) if one is bi-dialectal, Mandarin has to be one of their native dialects: (2) they can apply the 3rd tone sandhi to Mandarin nonce words (Zhang and Peng, 2013). For the assessment of (2), before EEG recording, each participant was asked to read out loud a list of 16 Mandarin disyllabic nonce words (order randomized), with four of them being underlyingly T3 + T3. At least one native Mandarin experimenter verified the application of the 3rd tone sandhi to the nonce words. All participants were right-handed. Two participants' data were excluded from further analysis due to the presence of a large number of artifacts (24.34% and 14.06%, respectively; see section EEG Processing for details). Of the remaining 29 participants whose data were analyzed, 16 also reported being a native speaker of another Chinese dialect.

### Design

The experimental blocks are summarized in Table 3. There are four blocks (A-D), each consisting of two sub-blocks (Original and Reversed), where the standard and the deviant are swapped. How the eight sub-blocks are related to the experimental conditions in Table 1 is also shown in Table 3. As a reminder, our study has a 2 ^*^ 2 design according to: (1) whether the standard and the deviant conflict at the UR tone level (UR-match vs. UR-mismatch); (2) whether the deviant is underlying T2 + T3 or T3 + T3 (Non-sandhi deviant vs. Sandhi deviant). The four conditions are: UR-match and Non-sandhi deviant (#1), UR-match and Sandhi deviant (#2), UR-mismatch and Non-sandhi deviant (#3), and UR-mismatch and Sandhi deviant (#4) (see Table 1).

**Table 3:** A summary of experimental blocks (the phonetic notations are IPA).

	**A (UR-match)**		**B (UR-mismatch)**		**C (UR-mismatch)**		**D (UR-match)**	
	**Standard**	**Deviant**	**Standard**	**Deviant**	**Standard**	**Deviant**	**Standard**	**Deviant**
Original	河马 /xə^2^ ma^3^/	皇马 /xuaŋ^2^ ma^3^/	河马 /xə^2^ ma^3^/	好马 /xaʊ^3^ ma^3^/	海马 /xai^3^ ma^3^/	皇马 /xuaŋ^2^ ma^3^/	海马 /xai^3^ ma^3^/	好马 /xaʊ^3^ ma^3^/
	UR-match and Non-sandhi deviant (#1)		UR-mismatch and Sandhi deviant (#4)		UR-mismatch and Non-sandhi deviant (#3)		UR-match and Sandhi deviant (#2)	
Reversed	皇马 /xuaŋ^2^ ma^3^/	河马 /xə^2^ ma^3^/	好马 /xaʊ^3^ ma^3^/	河马 /xə^2^ ma^3^/	皇马 /xuaŋ^2^ ma^3^/	海马 /xai^3^ ma^3^/	好马 /xaʊ^3^ ma^3^/	海马 /xai^3^ ma^3^/
	UR-match and Non-sandhi deviant (#1)		UR-mismatch and Non-sandhi deviant (#3)		UR-mismatch and Sandhi deviant (#4)		UR-match and Sandhi deviant (#2)	

*Each block (A–D) consists of the same stimuli, but the standards and the deviants are swapped in the sub-blocks (Original vs. Reversed). How these sub-blocks are related to the experimental conditions in Table 1 is also displayed*.

Näätänen and Kreegipuu (2011) emphasized the importance of using difference waves based on physically identical stimuli when isolating MMN. This was achieved by subtracting the response to a given stimulus when it was a standard from what was elicited by *the same* stimulus when it was a deviant (hence the term identity MMN). This way, low-level perceptual auditory responses to a specific stimulus can be better controlled. Our design allowed us to calculate identity MMN. In what follows, we use the term “identity difference wave” when the calculation of a difference wave uses physically identical stimuli.

### Procedure

Participants were instructed to attend passively to the auditory stimuli while watching a silent movie (*Rio*). Paired Original-Reversed sub-blocks were presented together; which one came first was counterbalanced across all participants. Among blocks A to D, the order was also counterbalanced across all participants. Within each sub-block, participants were presented with 400 stimuli, with 12.5% of them being deviants (thus, *n* = 50). All standards and deviants were randomly sampled from the corresponding stimulus set. The number of standards preceding a deviant varied from 5 to 9, with an equal probability of occurrence (20%) for each number. There were two additional constraints: (1) one standard stimulus cannot be repeated more than three times in succession; (2) no deviant is presented more than twice before another deviant is sampled. The above pseudo-randomization was achieved via a custom R script (R Core Team, 2020; version 3.5.1). Inter-stimulus intervals (ISI) were selected randomly between 700 ms and 900 ms (at a 50 ms increment). Including cap preparation, the entire session lasted ~2.5 h.

The EEG signal was recorded continuously using an elastic electrode cap with 32 Ag/AgCl scalp electrodes (Electro-Cap International, Inc.), arranged in a modified 10-20 layout (ground: AFz; midline: FPz, Fz, FCz, Cz, CPz, Pz, Oz; lateral: FP1/2, F3/4, F7/8, FT7/8, FC3/4, T3/4, C3/4, TP7/8, CP3/4, T5/6, P3/4, O1/2). Three bipolar montage electrode pairs (on the outer canthi, above and below each eye) were attached to monitor blinks and horizontal eye movements. One electrode was placed on each mastoid. Impedances for all scalp electrodes were kept below 5 kΩ. Paradigm (Tagliaferri, 2005; version 2.2.0.197) was used to control the delivery of the stimuli. During recording, data were sampled at 1 kHz and referenced to the left mastoid, amplified using a Neuroscan Synamps 2 amplifier (Compumedics Neuroscan, Inc.), and filtered with a bandpass of 0.1–200 Hz.

### EEG Processing

All offline EEG signal processing was carried out in EEGLAB (Delorme and Makeig, 2004; version 2019.1) and MATLAB (The MathWorks Inc.; version R2019b). The signal was first re-referenced to the average of both mastoid electrodes. Bad channels identified by either extreme distribution or poor connection during recording were interpolated. No more than five electrodes were interpolated for each participant. Continuous data were then epoched into −300 ms to 1200 ms intervals relative to the onset of each stimulus. Epochs were then decomposed into independent components using Independent Component Analysis by applying the *runica()* function in EEGLAB. For each participant, components that are typical of eye and muscle artifacts were first automatically identified by the ICLabel classifier (Pion-Tonachini et al., 2019). An experimenter verified and modified the automatically identified artifacts before removing them.

Data were then low-pass filtered at 30 Hz. Trials with extreme values (exceeding 100 μV in either direction), extreme range (having over 100 μV change in one trial), and extreme distribution (5 SDs away) were marked for rejection. If a trial contains more than three rejected electrodes, it was rejected. Otherwise, the bad electrodes within a trial were interpolated using the TBT EEGLAB extension (Ben-Shachar, 2020). Two participants with 24.34% and 14.06% trials being rejected were excluded from further analysis. Of the remaining 29 participants, each had fewer than 5% of trials rejected, with a minimum of 40 trials retained for each stimulus set in a sub-block. On average, each participant analyzed had 3.42% trials with at least one interpolated channel (SD = 1.22%). The remaining trials were then baseline corrected using the pre-onset 300 ms window. The recordings were then averaged in ERPLAB (Lopez-Calderon and Luck, 2014), taking into account the number of trials retained for each stimulus set.

ERPLAB was used to obtain point measurements from each participant. All electrodes were measured. There were two latency measures: 50% fractional pre-peak latency, 50% fractional area latency. The former is a more robust proxy of the onset latency, and the latter is a more reliable proxy of component latency than the peak latency (Luck, 2014). There were two amplitude measures: mean amplitude and signed area amplitude.^6^ The latter was used to provide a more precise characterization of a component when its polarity is taken into account (Luck, 2014). An interpolation factor of 2 was used for all measurements to diminish the influence of high-frequency noise.

### Statistical Analysis

Permutation-based analysis over the entire window of interest was performed (Maris and Oostenveld, 2007; Groppe et al., 2011a,b). The Mass Univariate ERP Toolbox (Groppe et al., 2011a; version 1.25.0.0) implemented in MATLAB (The MathWorks Inc.; version R2019b) was used to conduct such tests. Specifically, the cluster-based permutation test was applied to the difference waves to identify potential effects over relatively wide windows (lasting 150–200 ms). Electrodes within a distance of ~6.02 cm were considered as possible candidates for inclusion in a cluster, assuming the head's circumference was 56 cm. This configuration leads to an average of 3.1 neighboring channels for each electrode (SD = 0.9).

The input to the cluster-based permutation test^7^ was the difference wave obtained via calculating identity difference waves and other derivations (see the Results for details). A significant effect appeared as portions of the difference waves that deviated significantly from zero. The presence of an interaction was evaluated by comparing whether the difference between two difference waves differed significantly from zero. Unless otherwise specified, an alpha threshold of 0.05 was selected, and the number of permutations executed for each test was 5,000.

Whenever point measurements were obtained, linear mixed-effects modeling was employed to examine the effects of the independent variables. All models were fitted using the *lme4* package (Bates et al., 2015; version 1.1.21) in R (R Core Team, 2020; version 3.6.3). All mixed-effect models used restricted maximum likelihood estimation. Unless otherwise specified, all *post-hoc* comparisons and the corresponding *p*-values reported used the Bonferroni correction (Bonferroni, 1936). Whenever possible (no model convergence failure or singular fit), a random-slope of the key independent variable(s) was also included to avoid inflating the Type I error rate (Barr et al., 2013).

## Results

### Cluster-Based Permutation Tests

#### Is Segmental MMN Present in the First Syllable (S1)?

**Figure 3:**
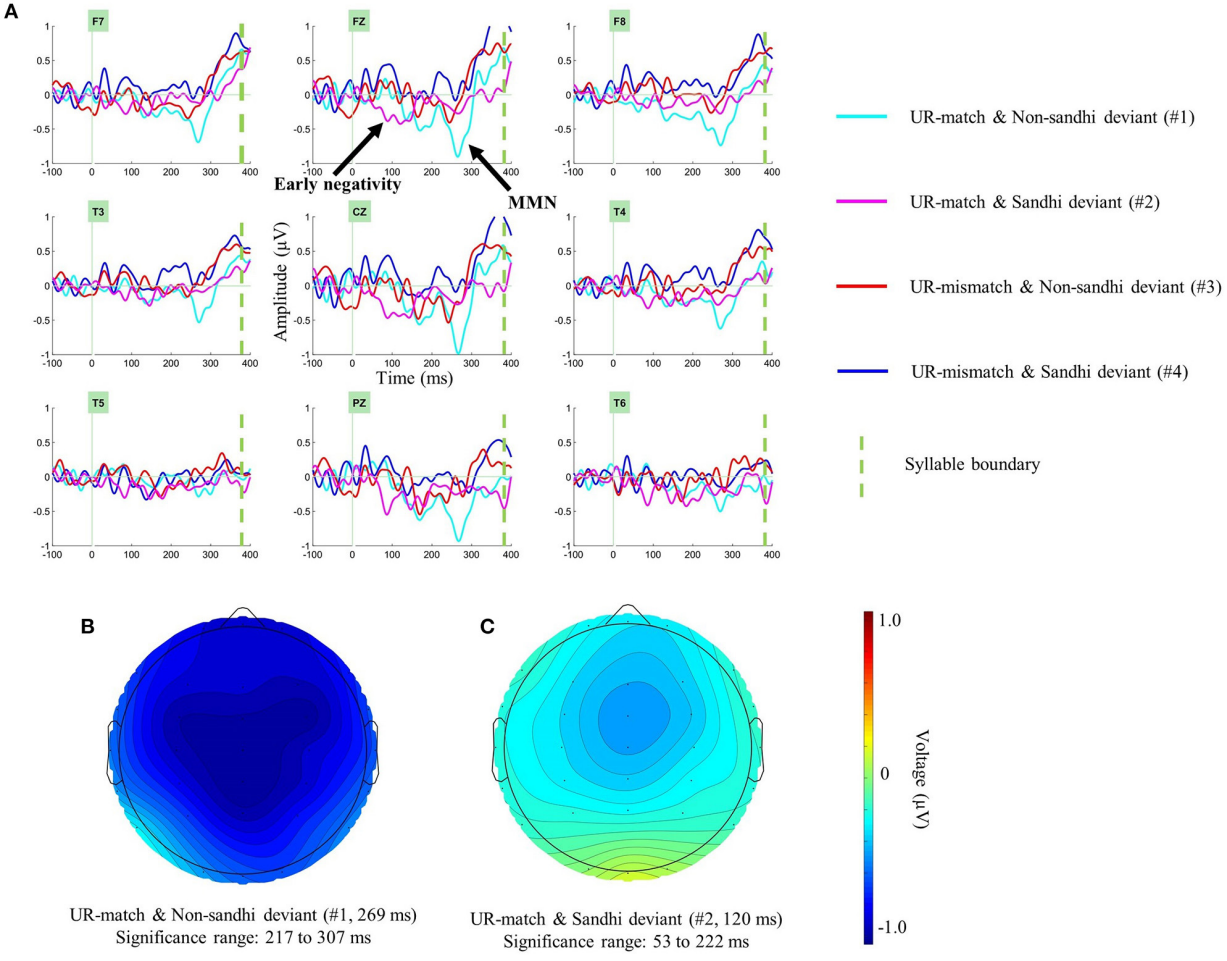
Examining the presence of segmental MMN in the first syllable (S1). Panel **(A)** shows identity difference waves (deviant minus standard) over selected electrodes in the first syllable. Panel **(B)** shows the topography of the significant negative cluster in the UR-match and Non-sandhi deviant Condition (#1), at a visually identified negative peak (269 ms). Panel **(C)** shows the significant negative cluster in the UR-match and Sandhi deviant Condition (#2), at a visually identified negative peak (120 ms). Only the negative cluster in the UR-match and Non-sandhi deviant Condition (#1) is interpreted as MMN.

The presence of segmental conflict led us to predict MMN in S1 in all conditions. We isolated identity difference waves for each condition (#1–#4), using the formulae in Table 4 (Segmental MMN in S1). When there is more than one formula in a cell, the difference waves in the same cell were collapsed. The right-most column in Table 4 contains a numeric index for each difference wave. In what follows, we use the difference wave index for brevity and clarity. Panel (A) of Figure 3 shows these four identity difference waves over selected electrodes. One-tailed cluster-based permutation tests were applied to evaluate the presence of significant negativity in S1 (analysis window: 100–300 ms).

**Table 4:** A summary of the formula(e) for isolating difference waves.

**Analysis**	**Waveform label**	**Formula(e)**	**Index**
Segmental MMN in S1	UR-match and Non-sandhi deviant	Original A Deviant – Reversed A Standard	#1
		Reversed A Deviant – Original A Standard	
	UR-match and Sandhi deviant	Original D Deviant – Reversed D Standard	#2
		Reversed D Deviant – Original D Standard	
	UR-mismatch and Non-sandhi deviant	Original C Deviant – Reversed C Standard	#3
		Reversed B Deviant – Original B Standard	
	UR-mismatch and Sandhi deviant	Original B Deviant – Reversed B Standard	#4
		Reversed C Deviant – Original C Standard	
*UR Relation*	UR-mismatch – UR-match and Non-sandhi deviant	#3 – #1	#5
	UR-mismatch – UR-match and Sandhi deviant	#4 – #2	#6
*Deviant Sandhi Status*	UR-match and Sandhi deviant – Non-sandhi deviant	#2 – #1	#7
	UR-mismatch and Sandhi deviant – Non-sandhi deviant	#4 – #3	#8
UR Relation^*^ Deviant Sandhi Status	UR-mismatch – UR-match and Sandhi deviant – Non-sandhi deviant	#8 – #7 = #6 – #5 = (#4 + #1) – (#3 + #2)	#9

*The first column indicates the analysis performed and the corresponding section in Results. The second column includes a description of each difference wave. The column “Formula(e)” specifies how the difference waves are calculated: when there is more than one formula in a cell, the difference waves in the same cell were collapsed. The last column introduces an index for each difference wave, which facilitates discussion of the results*.

For the UR-match and Non-sandhi deviant Condition (#1), one significant negative cluster was found, ranging from 217 ms to 300 ms (*p* = 0.022). Because the upper bound of the analysis window (300 ms) was significant, an adjusted window (150–350 ms) was applied to better capture this cluster. This time, the significant cluster ranged from 217 ms to 307 ms (*p* = 0.025). Panel (B) of Figure 3 shows the topography of this negativity at a visually identified negative peak (269 ms).

Another significant negative cluster was found in the UR-match and Sandhi deviant Condition (#2), ranging from 100 ms to 222 ms (*p* = 0.01). Because the lower bound of the analysis window (100 ms) was significant, an adjusted window (50–250 ms) was applied. This time, the significant cluster ranged from 53 ms to 222 ms (*p* = 0.003). Panel (C) of Figure 3 shows the topography of this negativity at a visually identified negative peak (120 ms).

Of these two negativities, only the negativity in #1 resembles MMN, peaking at ~250 ms after the onset of the deviant^8^. The latency characteristics of the negative cluster in #2 (onset: 53 ms; peak: 120 ms) made it unlikely to represent MMN (Näätänen et al., 2007). There was no other significant cluster (*p*-values > 0.31).

In summary, we only found convincing evidence for S1 segmental MMN in one condition: the UR-match and Non-sandhi deviant Condition (#1).

#### The Effect of *UR Relation*

To explore the effect of the independent variable *UR Relation* (UR-match vs. UR-mismatch) across time (i.e., S1 and S2 included), another two identity difference waves (#5 and #6) were calculated. Their formulae are summarized in Table 4 (*UR Relation*). A significant *UR Relation* effect is mathematically equivalent to regions of #5 and #6 that diverged significantly from zero. These two identity difference waves were submitted to two-tailed cluster-based permutation tests, using 150 ms windows (analysis windows: 0–150 ms, 150–300 ms, 300–450 ms, 450–600 ms, 600–750 ms, 750–900 ms). Table 5 summarizes the significant clusters found, together with the range of the cluster, the polarity of the cluster, and the position of a visually identified peak within a specific cluster. An empty cell in Table 5 indicates non-significance.

**Table 5:** A summary of significant clusters found in the identity difference waves #5 and #6, examining the effect of *UR Relation*.

**Analysis window (ms)**	**#5 (UR-mismatch – UR-match and Non-sandhi deviant)**			**#6 (UR-mismatch – UR-match and Sandhi deviant)**		
	**Range (ms)**	**Polarity**	**Peak (ms)**	**Range (ms)**	**Polarity**	**Peak (ms)**
0–150		(*p* > 0.38)		63–114	Positive (*p* = 0.025)	85
150–300	224–300 (224–376)	Positive (*p* = 0.012)	276		(*p* > 0.72)	
300–450		(*p* > 0.06)		300–396 (292–396)	Positive (*p* = 0.014)	350
450–600		(*p* > 0.06)			(*p* > 0.99)	
600–750	600–705 (554–705)	Positive (*p* = 0.013)	600		(*p* > 0.31)	
750–900		(*p* > 0.55)			(*p* > 0.50)	

*Only the range and peak of significant clusters are included^a^*.

*^a^The windows in brackets were included because the original cluster was significant at the boundary, and a follow-up analysis was attempted. For example, one significant cluster in #5 is from 224 ms to 300 ms. As the upper bound of this cluster was significant, an adjusted window of 200–400 ms was applied. The new significant cluster ranged from 224 ms to 376 ms (p = 0.005), which is shown in brackets*.

**Figure 4:**
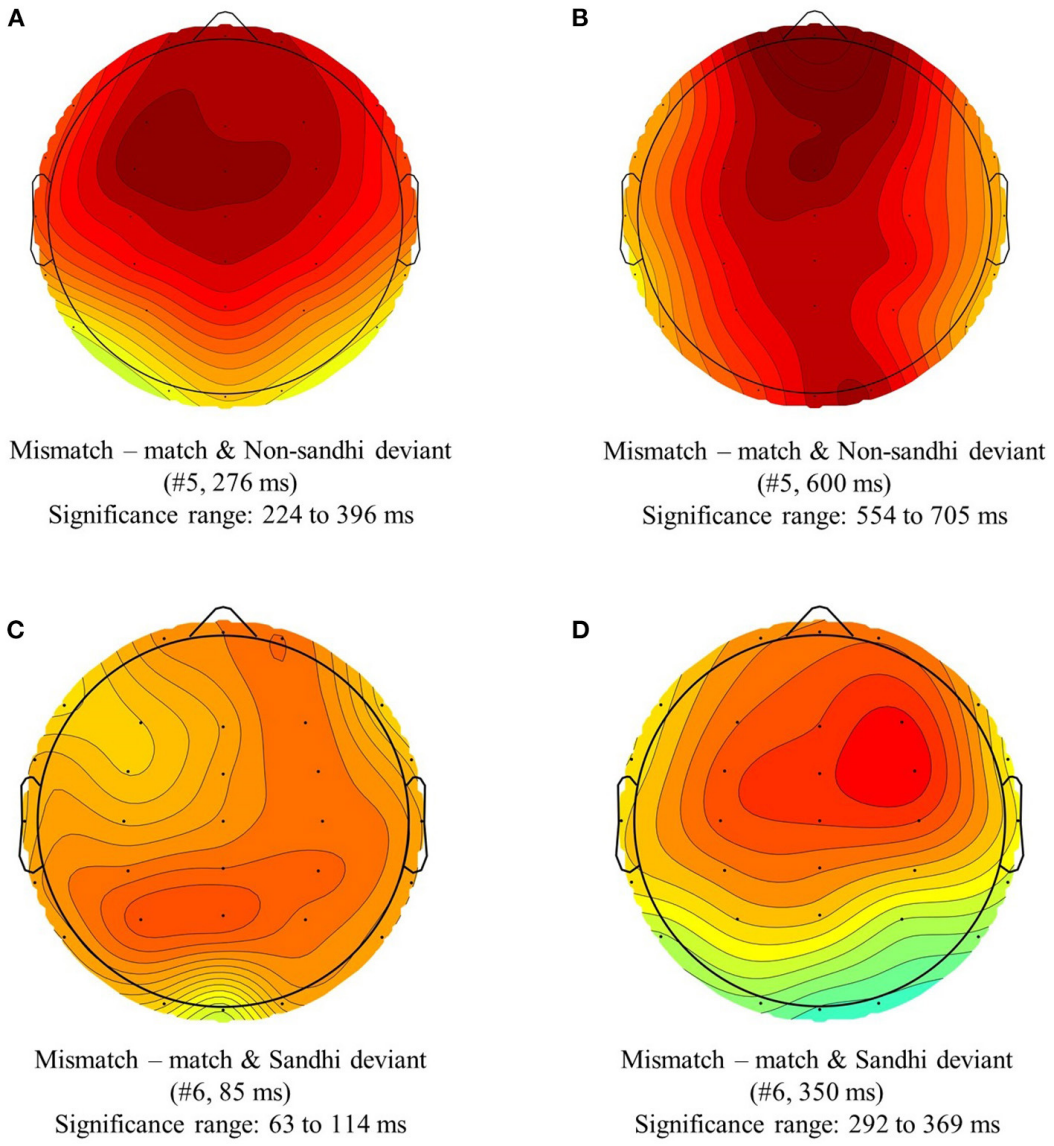
Topographic distribution of the significant positive clusters found in the waveforms Mismatch—match and Non-sandhi deviant (#5), and Mismatch—match and Sandhi deviant (#6), at visually identified positive peaks. Panel **(A)** shows a positive cluster in #5 (Mismatch—match and Non-sandhi deviant), peaked at 276 ms, and ranged from 224 ms to 396 ms. Panel **(B)** shows another positive cluster in #5 (Mismatch—match and Non-sandhi deviant), peaked at 600 ms, and ranged from 554 ms to 705 ms. Panel **(C)** shows a positive cluster in #6 (Mismatch—match and Sandhi deviant), peaked at 85 ms, and ranged from 63 ms to 114 ms. Panel **(D)** shows another positive cluster in #6 (Mismatch—match and Sandhi deviant), peaked at 350 ms, and ranged from 292 ms to 369 ms.

We found two significant positive clusters in #5, ranging from 224 ms to 396 ms, and from 554 ms to 705 ms, respectively. We also found two significant positive clusters in #6, ranging from 63 ms to 114 ms, and from 292 ms to 396 ms, respectively. Figure 4 plots these clusters at visually identified peaks of these positive clusters.

In summary, the independent variable *UR Relation* (UR-match vs. UR-mismatch) modulated the ERP responses in both S1 and S2. In all significant findings, the UR-mismatch conditions are more positive than the UR-match conditions.

#### The Effect of *Deviant Sandhi Status*

Another two difference waves (#7 and #8) were calculated to evaluate whether the independent variable *Deviant Sandhi Status* modulated the ERP responses. Their formulae are summarized in Table 4 (*Deviant Sandhi Status*). A significant effect of *Deviant Sandhi Status* is mathematically equivalent to regions of #7 and #8 deviating significantly from zero. Note that when calculating #7 and #8, ERP responses to physically non-identical stimuli were used, so #7 and #8 are not identity difference waves. The use of difference waves to probe cognition pre-supposes latency-aligned processes. It is highly likely that our stimuli differ in their recognition point and disambiguation point^9^. Consequently, a *Deviant Sandhi Status* effect using non-identity difference waves should be interpreted cautiously, especially in S1, where segmental conflict is also present.

The non-identity difference waves #7 and #8 were submitted to two-tailed cluster-based permutation tests, using 150 ms windows (analysis windows: 0–150 ms, 150–300 ms, 300–450 ms, 450–600 ms, 600–750 ms, 750–900 ms). No significant cluster was found (*p*-values > 0.091). Therefore, we did not find latency-aligned *Deviant Sandhi Status* effects with the cluster-based permutation tests.

#### The Interaction Between *UR Relation* and *Deviant Sandhi Status*

The *UR Relation* ^*^ *Deviant Sandhi Status* interaction can be evaluated by testing whether the difference between #5 and #6, together with the difference between #7 and #8, diverges significantly from zero. Because #5 = #3 − #1, #6 = #4 − #2, #7 = #2 − #1, and #8 = #4 − #3, the newly calculated difference wave, labeled #9, equals #8 − #7 = #6 − #5 = (#4 + #1) − (#3 + #2). The formula for calculating #9 is also included in Table 4 (*UR Relation* ^*^ *Deviant Sandhi Status*). Note that #9 is not an identity difference wave, so the issue of latency-alignedness remains. The non-identity difference wave #9 was submitted to two-tailed cluster-based permutation tests, using 150 ms windows (analysis windows: 0–150 ms, 150–300 ms, 300–450 ms, 450–600 ms, 600–750 ms, 750–900 ms). No significant cluster was found (*p*-values > 0.08). Therefore, the cluster-based permutation tests did not find evidence for a *UR Relation* ^*^ *Deviant Sandhi Status* interaction.

### Analysis Over Point Measurements

**Figure 5:**
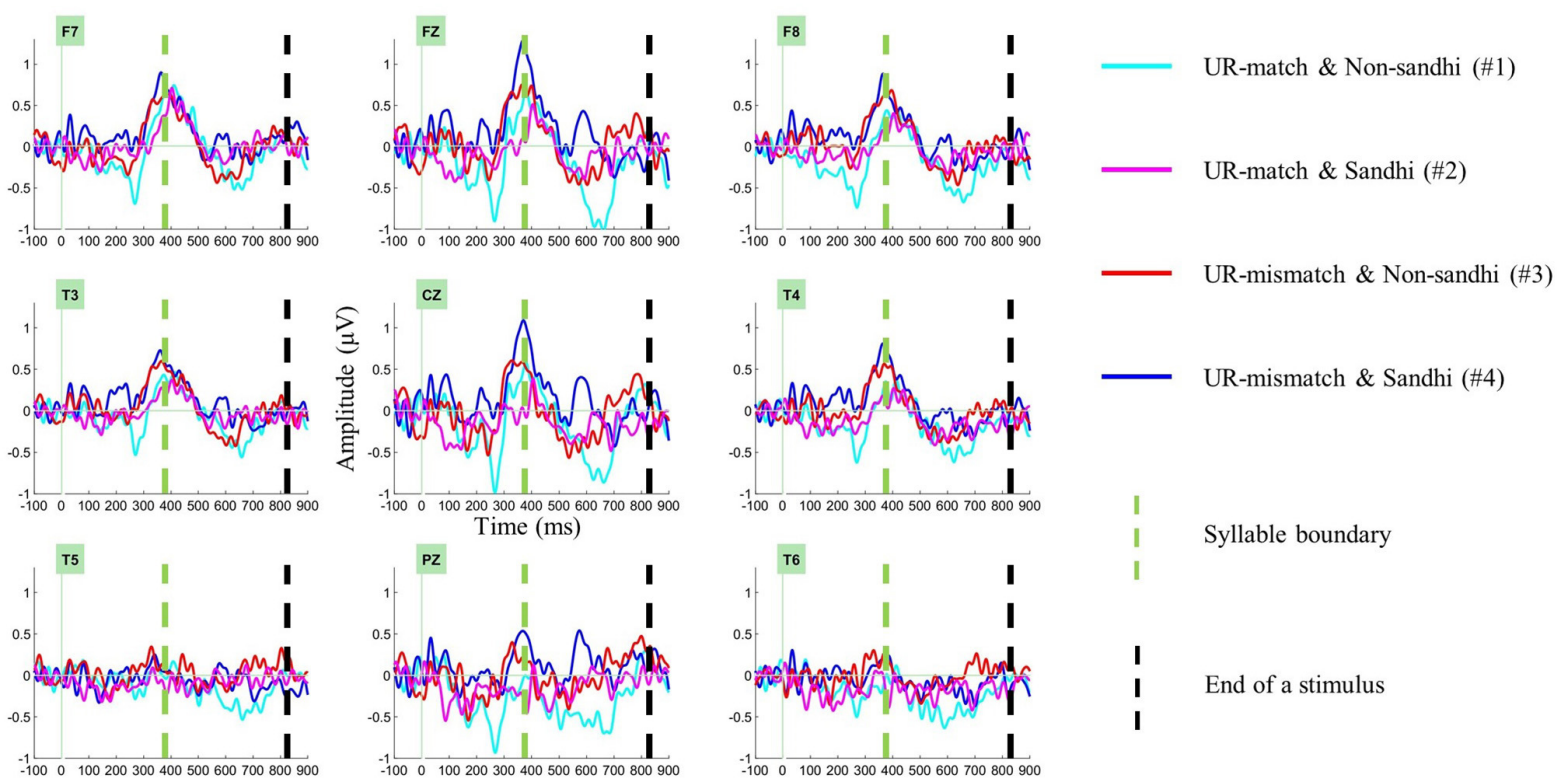
Identity difference waves (deviant minus standard) for all conditions over selected electrodes. The waveforms at the syllable boundary are either positive-going or positive, followed by negative-going waves in the second syllable (S2), suggesting that the S2 negativity cannot be explained by spill-over effects from the first syllable (S1).

This section reports how our independent variables and their interaction modulate point measurements obtained from the four identity difference waves (#1–#4). Figure 5 plots these identity difference waves of the experimental conditions across the two syllables over selected electrodes. According to visual inspection, these identity difference waves are characterized by a positive peak near the S1-S2 syllable boundary and a negative peak in the middle of S2. We obtained point measurements from the S1-S2 transitional positivity and the S2 negativity. In light of the range of significant clusters reported in Table 5, the measurement windows were set to 250–400 ms and 550–700 ms, respectively.

#### S1–S2 Transitional Positivity

**Figure 6:**
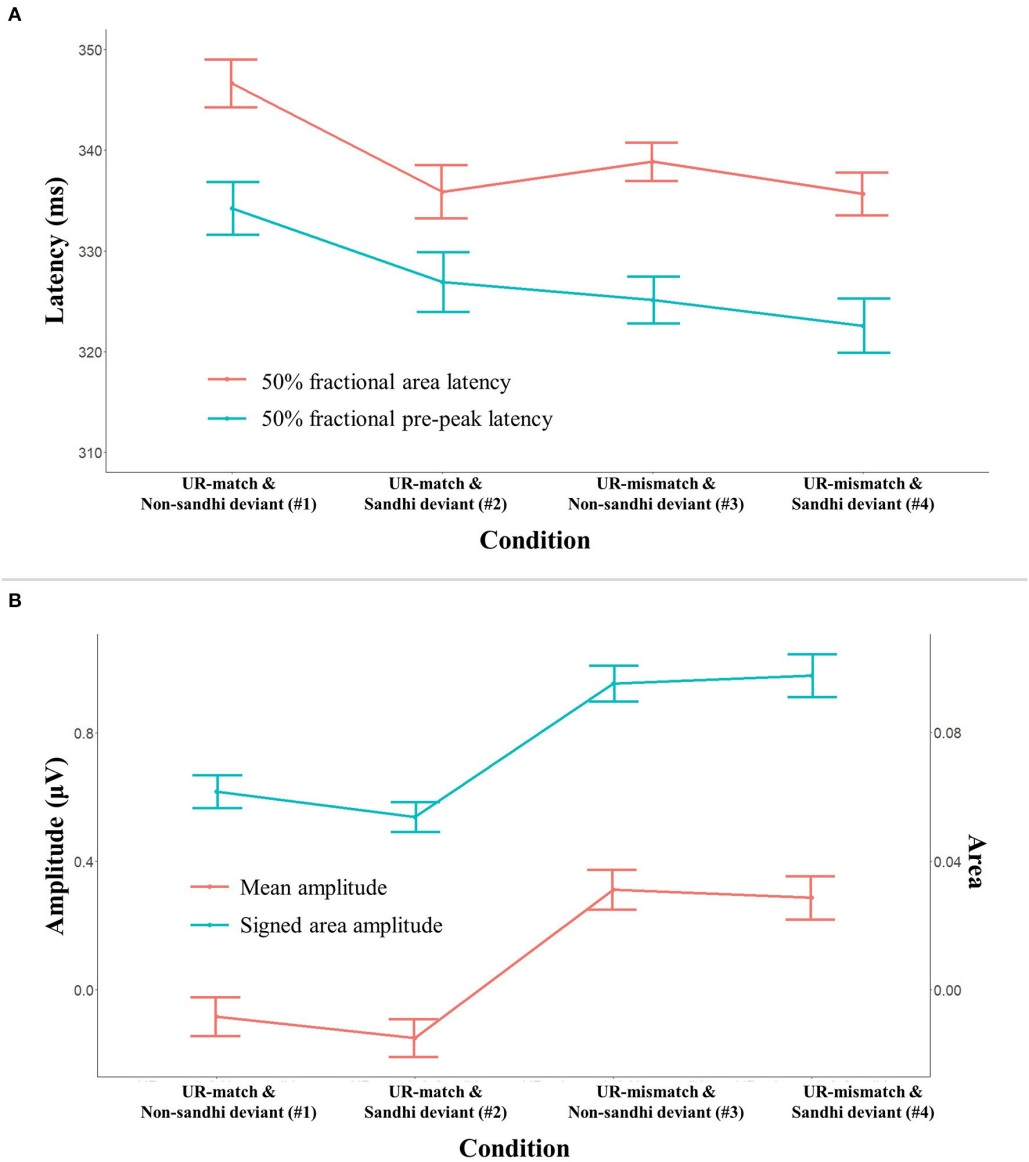
Latency (upper panel) and amplitude (lower panel) measures for the S1-S2 transitional positivity. The error bars indicate 95% confidence interval (95% CI). Panel **(A)** shows two latency measures (50% fractional area latency, 50% fractional pre-peak latency) as a function of experimental condition. Panel **(B)** shows two amplitude measures (mean amplitude, signed area amplitude) as a function of experimental condition.

Panel (A) of Figure 6 plots two latency measures (50% fractional pre-peak latency and 50% fractional area latency) for all conditions (#1–#4). The error bars indicate 95% confidence interval (95% CI). Mixed-effects modeling found a main effect of *Deviant Sandhi Status* for both latency measures (*p*-values <0.002): the Sandhi deviant conditions had earlier latency than the Non-sandhi deviant conditions (50% fractional pre-peak latency = 324.88 ms, 329.84 ms, respectively; 50% fractional area latency = 335.89 ms, 343.12 ms, respectively). There was also a main effect of *UR Relation* (*p*-values < 0.001): the UR-mismatch conditions had earlier latency than the UR-match conditions (50% fractional pre-peak latency = 323.94 ms, 330.78 ms, respectively; mean 50% fractional area latency = 337.42 ms, 341.59 ms, respectively). The interaction *UR Relation* ^*^ *Deviant Sandhi Status* was also significant for both measures (*p*-values < 0.05).

Panel (B) of Figure 6 plots two amplitude measures (mean amplitude and signed area amplitude) across all conditions (#1–#4). Mixed-effects modeling found a main effect of *UR Relation* for both amplitude measures (*p*-values < 0.001): the UR-mismatch conditions are more positive than the UR-match conditions (mean amplitude = 0.30 μV, −0.13 μV, respectively). The main effect of *Deviant Sandhi Status* was non-significant for both measures (*p*-values > 0.21). The *UR Relation* ^*^ *Deviant Sandhi Status* interaction was significant for the signed area amplitude (*p* = 0.041). No other effect was significant.

#### S2 Negativity

**Figure 7:**
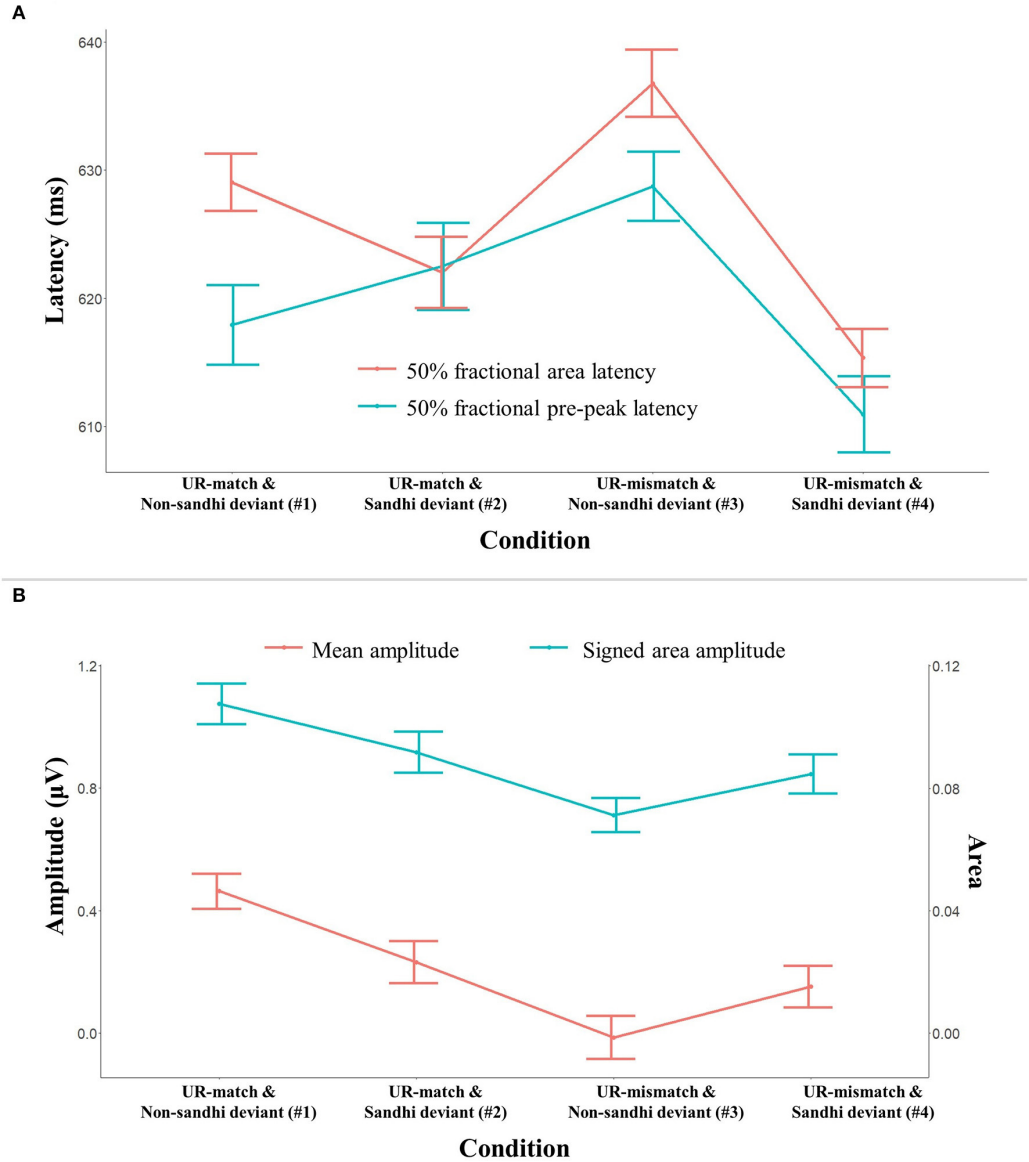
Latency (upper panel) and amplitude (lower panel) measures for the S2 negativity. The error bars indicate 95% confidence interval (95% CI). Panel **(A)** shows two latency measures (50% fractional area latency, 50% fractional pre-peak latency) as a function of experimental condition. Panel **(B)** shows two amplitude measures (mean amplitude, signed area amplitude) as a function of experimental condition.

Panel (A) of Figure 7 plots the latency measures for the S2 negativity from all conditions (#1–#4). The error bars indicate 95% confidence interval (95% CI). Mixed-effects modeling found a main effect of *Deviant Sandhi Status* for both latency measures (*p*-values < 0.001): the Sandhi deviant conditions had earlier latency than the Non-sandhi deviant conditions (mean 50% fractional pre-peak latency = 616.30 ms, 623.12 ms, respectively; mean 50% fractional area latency = 618.43 ms, 632.97 ms, respectively). The main effect of *UR Relation* was non-significant for either measure (*p*-values > 0.57). A significant *UR Relation* ^*^ *Deviant Sandhi Status* interaction was found for both latency measures (*p*-values < 0.001).

Panel (B) of Figure 7 plots the amplitude measures from all conditions (#1–#4): the amplitude is presented in absolute values, so that the size comparison across conditions is more straightforward. Mixed-effects modeling found a main effect of the *UR Relation* for both amplitude measures (*p*-values < 0.001): the UR-mismatch conditions are less negative than the UR-match conditions (mean amplitude: −0.35 μV, −0.73 μV, respectively). The two amplitude measures also returned a significant main effect of *Deviant Sandhi Status* (*p*-values < 0.035): overall, the negativity in the Non-sandhi deviant conditions was stronger (mean amplitude = −0.24 μV, −0.18 μV, respectively). Finally, both amplitude measures yielded a significant *UR Relation* ^*^ *Deviant Sandhi Status* interaction (*p*-values < 0.001).

Overall, our two independent variables and their interaction modulated the timing and the amplitude of the S1-S2 transitional positivity and the S2 negativity.

## Discussion

Our study examined the process of perceiving an alternated sound and the electrophysical signatures associated with this process, using the Mandarin 3rd tone sandhi as a test case. The results include a complex set of significant findings throughout the stimulus, including at S1, the S1-S2 transitional region, and S2. In what follows, we first scrutinize whether these significant findings could be attributed to the relationships between standards and deviants manipulated in our experimental design (the segmental MMN, effects of *UR Relation* and *Deviant Sandhi Status*). Next, we interpret the results from a broader perspective and relate the findings to our research questions (the process of perceiving an alternated sound and the electrophysical signatures associated with this process). Whether the brain can access the UR of alternated sounds in a passive-listening experiment is first discussed. The mechanisms underlying the process of perceiving alternated sounds and the potential corresponding electrophysiological signatures are also discussed. A tentative model for the process that may account for our results is proposed. Possible future research avenues, including the testing of the proposed model, are also briefly commented on.

### Can the Significant Results Be Attributed to Our Experimental Design?

#### Segmental MMN

##### Segmental MMN Is Observed Only When There Is Neither Indirect SR-UR Mapping Nor UR-Mismatch

There is a segmental conflict in all experimental conditions in S1, which should have yielded MMN under normal circumstances. However, we only found two significant negative clusters in two conditions: the UR-match and Non-sandhi deviant Condition (#1), the UR-match and Sandhi deviant Condition (#2).

We only interpret the negative cluster in the UR-match and Non-sandhi deviant Condition (#1) as MMN. The negative cluster in the UR-match and Sandhi deviant Condition (#2) is unlikely to be MMN. On the one hand, its latency characteristics (onset: 53 ms; peak: 120 ms) is earlier than what is typical for MMN (Näätänen et al., 2007; Näätänen, 2008). Also, consider the lexical items presented in the UR-match and Non-sandhi deviant Condition (#1): /xə^2^ ma^3^/ and /xuaŋ^2^ ma^3^/. These two lexical items disambiguate earlier than the two lexical items in the UR-match and Sandhi deviant Condition (#2) (/xaʊ^3^ ma^3^/ and /xai^3^ ma^3^/). If we observe MMN in #2, its onset latency should be later than #1. In reality, our data shows that the negativity in #2 occurs earlier, rather than later than in #1.

Is it possible that the later portion of the negative cluster in #2 contains MMN? We do not think so. The negative cluster in #1, which contains MMN, ranges from 217 ms to 307 ms; in contrast, the negative cluster in #2 ranges from 53 ms to 222 ms. The less drastic segmental conflict in #2 should have led to MMN later than what is observed in #1. Though the upper bound of the negative cluster in #2 (222 ms) is slightly later than the lower bound of the negative cluster in #1 (217 ms), the difference is minuscule (5 ms), especially when compared with the duration of the negative cluster in #2 (169 ms). Therefore, even if the negative cluster in #2 contains traces of MMN, its influence should be negligible.

What is intriguing, however, is the origin of the early negativity in #2. Given its early onset latency (53 ms) and relatively long duration (169 ms), it might not reflect only the physical properties of the stimuli; it might reflect more than one processing operation as well. Compared to the two other conditions where MMN is also absent (UR-mismatch and Non-sandhi deviant, #3; UR-mismatch and Sandhi deviant, #4), #2 (UR-match and Sandhi deviant) does not have an underlying tone conflict. In other words, the early negativity in #2 is free from the interference of UR-mismatch. Therefore, indirect SR-UR mapping is one candidate explanation for the early onset of the negativity in #2. In #2, both the standards and the deviants are underlying T3 + T3 that involves indirect SR-UR mapping. Future research is needed to illuminate how the early negativity in #2 is related to indirect SR-UR mapping.

In summary, despite the apparent segmental conflict in S1 in all conditions, we only found convincing evidence for segmental MMN in the UR-match and Non-sandhi Condition (#1). In this condition, both the standards and the deviants are underlying T2 + T3, permitting a direct SR-UR mapping. Moreover, there is no UR-mismatch in this condition. Stated differently, segmental MMN only emerges when circumstances are the most favorable (no UR-mismatch, and no need for extra processing to arrive at the correct UR). That an MMN is only clearly observable in the UR-match and Non-sandhi Condition (#1) provides indirect evidence for the SR-UR mapping process: UR-mismatch or extra effort to arrive at the correct UR, or a combination of these two mechanisms, could interfere with the normal generation of the predicted segmental MMN. Future research is necessary to clarify the nature of this interference on normal MMN generation: segmental MMN could have been generated but masked (a masking account for the disappearance of MMN); alternatively, the normal generation MMN could have been interrupted due to more effortful processing of the UR (an interruption account for the disappearance of MMN).

##### Underspecification Cannot Explain the Observed Pattern

Before moving on, it is important to consider the influence of underspecification, as our test case involves Mandarin T3, which has been hypothesized to be underspecified. Proper discretion should be exercised to avoid mistaking the effect of underspecification for the effect of SR-UR mapping. Specifically, when comparing T3 + T3 deviants and T2 + T3 deviants, which have identical surface tone realizations, if we observe an MMN asymmetry (i.e., T2 + T3 deviants showing greater MMN than T3 + T3 deviants), we could attribute this MMN asymmetry to T3 being underspecified; alternatively, we could interpret the T3 + T3 deviants' reduced MMN amplitude as an indication of its more effortful SR-UR mapping process.

Our results show that neither the UR-mismatch and Non-sandhi deviant (#3) Condition nor the UR-mismatch and Sandhi deviant (#4) Condition yielded MMN. Based on visual inspections of Panel (A) of Figure 3, the UR-mismatch and Non-sandhi Condition (#3) is more positive, thus less negative, than the UR-mismatch and Sandhi deviant Condition (#4), which is consistent with an underspecification account (i.e., the UR-mismatch and Sandhi deviant Condition (#4) being more negative). We conducted a series of tests on the difference between #3 and #4 in S1. The most powerful is a one-tailed cluster-based permutation test with a 50 ms window size (range examined: 150–300 ms). None of the tests yielded a significant result (*p*-values > 0.10), suggesting that the difference between #3 and #4 in Panel (A) of Figure 3 is only numerical. Therefore, we conclude that our data cannot be explained by underspecification.

#### The Effect of *UR Relation*

##### Interpreting the Significant Positive Clusters: Not All Significant Positive Clusters Can Be Attributed to UR-Mismatch

All the positive clusters found by the permutation tests suggest that the UR-mismatch conditions are more positive than the UR-match conditions (cf. section The effect of *UR Relation*; Table 5; Figure 4). Given such uniform findings, one may be tempted to conclude that the UR-mismatch conditions were consistently more positive than the UR-match conditions from the beginning to the end of the deviants, and these positive clusters indicate a consistent UR-mismatch effect. However, a close inspection of these positive clusters revealed different causes: not all significant positive clusters can be attributed to UR-mismatch.

One apparent spurious cluster is the first positive cluster in #6 (Mismatch–match and Sandhi deviant), centered around 85 ms (Panel (C) of Figure 4). This positive cluster was mainly driven by the negative cluster in the UR-match and Sandhi deviant Condition (#2) (cf. Panel (C) of Figure 3): because #6 = #4 – #2, this positive cluster is an outcome of subtracting a negative cluster from zero. As discussed previously in section Segmental MMN, the origin of the early negativity in #2 is unclear. However, its early onset latency (53 ms) made it unlikely to be attributable to *UR Relation*: listeners are still perceiving the fricative /x/ at 53 ms post-onset. The two lexical items in the UR-match and Sandhi deviant Condition also have similar fricatives due to their identical nuclear vowel (/xaʊ^3^ ma^3^/ and /xai^3^ ma^3^/). Consequently, listeners could not have identified the UR of the deviant as early as 53 ms in the UR-match and Sandhi deviant Condition (#2). The identity difference wave #5 (UR-mismatch–UR-match and Non-sandhi deviant) did not show a positivity this early, either: a genuine UR-mismatch effect should be present for both the Sandhi deviant (#5) and the Non-sandhi deviant (#6). Therefore, we do not interpret this early positivity in #6 (Mismatch–match and Sandhi deviant) as an effect of *UR Relation*.

At the S1-S2 transitional region (from ~300 ms to 400 ms; see Panels (A) and (D) of Figure 4), both #5 and #6 yielded a significant positive cluster. According to Figure 5, responses across all conditions in this S1-S2 transitional region were positive-going. This means that the positive clusters we found in #5 and #6 in the S1-S2 transitional region reflect the differential magnitude of the positive-going waves (#1–#4) in Figure 5: in addition to overall positive-going waveforms in the S1-S2 transitional region, UR-mismatch conditions are more positive than UR-match conditions. We interpret the two positive clusters found in the S1-S2 transitional region in #5 and #6 as an effect of *UR Relation*, as it is present for both the Sandhi deviant (#5) and the Non-sandhi deviant (#6).

We also found a positive cluster in the S2 of #5 (Mismatch–match and Non-sandhi deviant), centered around 600 ms. The identity difference wave #6 (Mismatch–match and Sandhi deviant) did not yield a positive cluster within a similar time window. To evaluate whether the null finding in #6 might have been a Type II error, another one-tailed cluster-based permutation test was applied to #6 (analysis window: 650 ms to 800 ms). No significant cluster was found (*p* = 0.14). Thus, we only found a positive cluster in the S2 of #5 (Mismatch–match and Non-sandhi deviant). In light of the waveforms in Figure 5, this positive cluster should reflect the differential behavior of the S2 negativity. Our analysis of the point measurements from the S2 negativity found a main effect of *UR Relation*: the UR-mismatch conditions are less negative than the UR-match conditions (cf. section S2 Negativity). *Post-hoc* comparison also did not find a significant difference between the UR-mismatch and Sandhi deviant (#4) and the UR-match and Sandhi deviant (#2) (*p* = 0.11; cf. Figure 7) for both amplitude measures from the S2 negativity, suggesting that for Sandhi deviants, the UR-mismatch condition is not more positive (thus, less negative) than the UR-match condition.

However, a significant *UR Relation* ^*^ *Deviant Sandhi Status* interaction was found for both amplitude measures, suggesting the influence of *Deviant Sandhi Status*. That is, the positive cluster in the #5 (Mismatch–match and Non-sandhi deviant) may be attributable to *UR Relation*. The influence of *Deviant Sandhi Status* in #6 (Mismatch–match and Sandhi deviant; indirect SR-UR mapping) might have masked the more positive effect of *UR Relation* in S2, if it indeed is present. What is critical is the processing mechanisms in S2, which are unclear for the moment. Future research is needed before one can definitively state whether the UR-mismatch conditions are more positive than the UR-match conditions in S2.

In summary, the most convincing evidence for an effect of *UR Relation* is found at the S1-S2 transitional region: UR-mismatch conditions are more positive than UR-match conditions at this region, and this is true for both the Non-sandhi deviants and the Sandhi deviants.

##### P300 and the S1-S2 Transitional Positivity

The polarity and the timing of the S1-S2 transitional positivity in #5 and #6 are also reminiscent of another extensively studied ERP component: P300 (Polich and Kok, 1995; Polich, 2003, 2007, 2012). Although P300 is usually observed in studies with an overt task, P300 has also been reported in passive-listening MMN experiments when the deviant differs sufficiently from the standards (see Friedman et al., 2001, for a review). Specifically, P300 elicited in passive-listening MMN studies has been classified as P3a and linked to involuntary attention allocated to a deviant (Friedman et al., 2001; Paavilainen, 2013). Recently, Gansonre et al. (2018) also reported P300-like effects in a passive-listening MMN experiment using speech stimuli.

We do not interpret the S1-S2 transitional positivity as P3a for two reasons. First, MMN is absent in three out of the four conditions. In a passive-listening MMN experiment, it is unclear whether P3a will appear when MMN is absent. Second, when the original ERP waveforms, rather than the identity difference waves, are plotted, it is clear that P300 alone cannot explain the positivity in the S1–S2 transitional positivity (see Supplementary Figure 1 for details; the window for P300 is shaded in gray). Therefore, we conclude that the S1-S2 transitional positivity as an effect of *UR Relation* is not equivalent to P300.

#### The Effect of *Deviant Sandhi Status*

Cluster-based permutation tests did not find a main effect of *Deviant Sandhi Status*, nor a *UR Relation* ^*^ *Deviant Sandhi Status* interaction. The independent variable *Deviant Sandhi Status* was significant in some of the point measurements. Specifically, in the S1-S2 transitional region, the Sandhi deviant conditions had earlier latency than the Non-sandhi deviant conditions; this is also true in S2. The chance of both findings being driven by the physical properties of the stimuli is slight. For one thing, in the S1, the Non-sandhi deviants should not have later disambiguation points than the Sandhi deviants, since the /xai^3^ ma^3^/ and /xaʊ^3^ ma^3^/ pair (both being Sandhi deviants) is the hardest to disambiguate among all standard-deviant pairs. For another, though not physically identical, all stimuli have the same S2 (/ma^3^/). Thus, we tentatively interpret that the Sandhi deviant had earlier latency than the Non-sandhi deviant at the S1-S2 transitional region and in S2 is an effect of *Deviant Sandhi Status*.

### Interpreting the Significant Findings From a Broader Perspective

#### Can the UR of an Alternated Sound Be Accessed in a Passive-Listening MMN Experiment?

Our answer to this question is yes: the UR of an alternated sound can be accessed in a passive-listening MMN experiment.

Since its discovery, MMN has shown its sensitivity to multiple levels of representations in the brain, ranging from sensory responses to physical properties of the stimuli (Sams et al., 1985; Paavilainen et al., 2003) to arbitrarily formed abstract rules (Paavilainen et al., 2001; Korzyukov et al., 2003; Schröger et al., 2007). For language, investigations into various aspects of abstraction have also been attempted with MMN, including phonological features such as [± coronal] (Lahiri and Reetz, 2010; Højlund et al., 2019) and syntactic properties such as gender agreement (Hasting et al., 2007; Pulvermüller and Assadollahi, 2007; Hanna et al., 2017). Another emerging line of research examines the MMN's sensitivity to the lexical properties of individual stimulus tested (Alexandrov et al., 2011; Shtyrov et al., 2011; Hanna et al., 2017). With the accumulation of replication using multiple lexical items and testing various languages, the field is growing increasingly confident that MMN could access individual lexical items and their linguistic properties, such as phonotactic probability (Steinberg et al., 2009, 2010, 2011), frequency (Alexandrov et al., 2011; Shtyrov et al., 2011) and semantic content (Shtyrov et al., 2004; Brunellière et al., 2011).

While most previous research does not distinguish SR and UR (for an exception, see Chien et al., 2020), our study contributes to the field by demonstrating access to UR in a passive-listening MNN experiment, even for an alternated sound involving neutralization. Our study differs from previous research in that we are not showing MMN latency/amplitude difference as a function of the representation under investigation. Instead, our results indicate that the SR-UR mapping process can interfere with the normal generation of MMN. In all conditions, there is an apparent segmental conflict between the standards and the deviants. However, MMN is only observed when there is a direct SR-UR mapping for both the standard and the deviant (i.e., when both are underlyingly T2 + T3). When there is a need for extra processing to uncover the correct UR, either for the standards, the deviants, or both, no MMN was found despite the obvious segmental conflict in S1.

What remains unclear, however, is the underlying mechanisms that led to the interference of the MMN in three out of the four experimental conditions. We proposed two possible explanations for the disappearance of MMN in our study: (1) a masking account, where MMN is still generated but masked by the processing of UR with an indirect SR-UR mapping; (2) an interruption account, where the normal generation MMN is interrupted due to more effortful processing of the UR. Future research is needed to distinguish these two accounts.

In addition to the absence of segmental MMN in conditions where it should have occurred, there are two other pieces of evidence for accessing the UR of the alternated T3 in our study: (1) an effect of *Deviant Sandhi Status*, which distinguishes T2 + T3 and T3 + T3 deviants, as the latter requires extra processing effort to achieve a correct SR-UR mapping; (2) an effect of *UR Relation*, which is contingent on the outcome of a correct SR-UR mapping.

In short, our study demonstrates that the pre-attentive processing of speech involves access to UR, even for a phonologically alternated sound involving neutralization. Access to UR can influence brain-level responses associated with surface-level segmental processing, as indexed by MMN in an oddball experiment. We hope that our results will encourage more neurolinguistic investigations into the electrophysiological signatures of perceiving alternated sounds, which is prevalent in language but vastly understudied.

#### The Process of Perceiving a Potentially 3rd Tone Sandhi Form (SR: T2 + T3)

##### Early Processing in S1

We found a significant negative cluster in the UR-match and Sandhi deviant Condition (#2) with an early onset latency (53 ms). In #2, before hearing the deviant, all standards are underlying T3 + T3 that involves more effortful SR-UR mapping. Moreover, the two lexical items in this condition (/xaʊ^3^ ma^3^/ and /xai^3^ ma^3^/) disambiguate relatively late. Therefore, the early onset of this negativity is unlikely to be driven by the physical properties of the stimuli. This early negativity may be related to the continuous perception of T3 + T3 standards. More research is needed to elucidate the underlying processing mechanism(s).

It is worth mentioning that although our data suggest that the influence of the UR emerges very early, at least in an MMN experiment, where there are substantial repetitions of the stimuli, it remains to be tested whether the influence of the UR of alternated sounds could show up this early during normal speech perception.

##### Accessing UR in S1 (Before S2)

Our working hypothesis assumes T2 as the default upon hearing a rising F0 contour. If the context suggests an alternative, then extra processing effort is exerted to arrive at the correct UR. No speech perception study has examined the time course of perceiving the Mandarin 3rd tone sandhi form. The closest example is Shen et al. (2013), which examined the time-course of perceiving monosyllabic Mandarin T2 and T3. Their results showed that listeners' eye movements are modulated by the acoustic properties of the stimuli: if the acoustic realization of the incoming sound is closer to a T2, the probability of looking at a T2 target is greater. It remains to be seen whether the acoustic realization of the S1 also modulates the perception of the 3rd tone sandhi, and whether treating T2 as the default parsing choice is a tenable position.

Our results also suggest that the perception of alternated sounds, like the perception of un-alternated sounds, is incremental. Recall that the effect of *UR Relation* occurs at the S1-S2 transitional region (before S2). This means that the brain does not wait until S2 is fully recognized before it starts to entertain the possibility of parsing S1 as an underlying T3. Pre-attentive learning and the development of expectations (Bendixen et al., 2012; Liu et al., 2018) in an MMN experiment with repeated exposure might explain the early onset of the *UR Relation* effect as well. After all, participants heard only two lexical items in one sub-block, and there were only four lexical items in our experiment (see the Methods section). We also conducted split-half analyses on our data, which compared the ERP responses in the first half and second half of each sub-block.

The detailed results of the split-half analysis are included in the Supplementary Material. In brief, in the S1-S2 transitional region, the positivity in the four identity waves (#1–#4) was stronger in the second half of each sub-block than in the first half (*p*-values < 0.001), suggesting the development of the positivity as the experiment progresses. Moreover, the two identity difference waves that directly tested the effect of *UR Relation* (#5 and #6) were also marginally significant at the S1-S2 transitional region (*p-*values < 0.08, two-tailed tests), showing that the UR-mismatch conditions were more positive than the UR-match conditions. Therefore, we propose that in our experiment, before hearing S2, the participants' brain started to access the UR of S1 in the S1-S2 transitional region, leading to UR-mismatch conditions being more positive than the UR-match conditions. It remains to be tested whether the UR of S1 can be accessed this early during normal speech perception, where the incoming stimuli are less likely to be repeated, thus less predictable.

##### Modeling the S1-S2 Transitional Positivity

In section S1-S2 Transitional Positivity, we reported how our two independent variables and their interactions modulated the latency and the amplitude of the S1-S2 transitional positivity. This raises the question of how these differences could have emerged. In the following, we attempt to provide an account.

In Figure 3, which plots the identity difference waves in all conditions (#1–#4), all waveforms in the S1-S2 transitional region were either positive or positive-going. Therefore, the S1-S2 transitional positivity reported as a *UR Relation* effect (based on #5 and #6) reflects the amplitude difference in the S1-S2 transitional region of #1–#4. Accordingly, we posit the existence of a “Base Positivity” in the S1-S2 transitional region of #1–#4. The origin and the function of this “Base Positivity” are unclear for the moment, calling for future studies.

**Figure 8:**
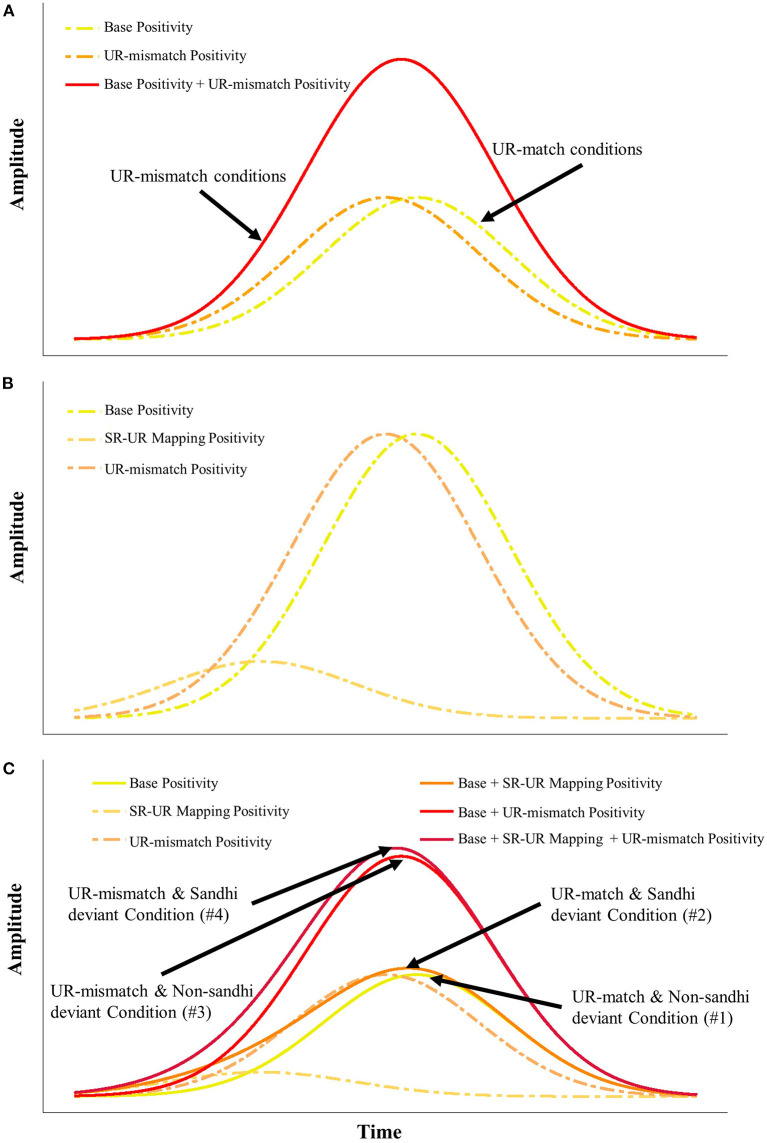
Potential processes involved in the S1-S2 transitional positivity as a function of the component summation. Panel **(A)** shows the modeling of the UR-mismatch conditions as a summation of Base Positivity and UR-mismatch Positivity. Panel **(B)** posits the presence of SR-UR Mapping Positivity (for the T3 + T3 deviants), which has an early peak but a smaller amplitude. Panel **(C)** illustrates how the summation of the three hypothetical components (Base Positivity, SR-UR Mapping Postitivty, UR-mismatch Positivity) could explain the latency and amplitude differences across conditions.

For UR-mismatch conditions, we posit the existence of an ERP component that indexes this UR-mismatch. Given that the UR-mismatch conditions had earlier latency and larger positivity than the UR-match conditions, we posit the existence of a “UR-mismatch Positivity,” whose peak latency should be earlier than the “Base Positivity.” This way, the UR-match conditions could be understood as reflecting the “Base Positivity,” while the UR-mismatch conditions could be viewed as a summation of the “Base Positivity” and the “UR-mismatch Positivity” (see Panel (A) of Figure 8).

We also hypothesize that sandhi deviants engage in extra processing due to indirect SR-UR mapping, and that there is an ERP component that reflects this operation (i.e., sandhi deviants should include this component, while non-sandhi deviants should not). Panel (B) of Figure 8 visualizes the relative timing and the amplitude of the hypothetical ERP component for indirect SR-UR mapping (i.e., “SR-UR mapping Positivity”), together with the “Base Positivity” and the “UR-mismatch Positivity.” The relative timing of the components involved is: SR-UR mapping (SR-UR Mapping Positivity) < UR-mismatch Positivity < Base Positivity. The order of the timing is based on two considerations: (1) the SR-UR mapping should precede decision about the *UR Relation*; (2) the UR-match and Non-sandhi (#1) Condition's latest latency could be attributed to the absence of the UR-mismatch. Notice the overlap in timing between the Mapping Positivity and the UR-mismatch Positivity in Panel (B). This echoes our assumption about incremental processing.

Moreover, in Panel (B) of Figure 8, the relative (peak) amplitudes of the components involved are: SR-UR mapping (SR-UR Mapping Positivity) < UR-mismatch Positivity = Base Positivity. The order and the directionality (e.g., the component for SR-UR mapping is positive) of the amplitudes are based on three considerations: (1) the main effect of *Deviant Sandhi Status* is non-significant for amplitude measures, suggesting the small amplitude of the SR-UR Mapping Positivity; (2) the signed area amplitude of the UR-mismatch conditions is approximately twice that of the UR-match conditions, suggesting similar amplitude of the UR-mismatch Positivity and the Base Positivity; (3) three of four conditions did not show MMN in the targeted window, which may contain an earlier portion of the SR-UR mapping component that is not modeled here—if the earlier portion of the SR-UR mapping component contributed to the disappearance of MMN, it should be positive.

Accordingly, Panel (A) can be updated to Panel (C) to highlight potential operations involved in each condition as a function of component summation: #1 = Base Positivity; #2 = Base Positivity + SR-UR Mapping Positivity; #3 = Base Positivity + UR-mismatch Positivity; #4 = Base Positivity + UR-mismatch Positivity + SR-UR Mapping Positivity. This way, we could explain the latency and amplitude findings reported in section S1-S2 Transitional Positivity for the S1-S2 transitional positivity with three hypothesized underlying mechanisms with distinct timing and amplitude profiles (SR-UR Mapping Positivity, UR-mismatch Positivity, and Base Positivity).

As noted by an anonymous reviewer, the SR-UR Mapping Positivity in the difference waves #2 and #4, in which the deviants are sandhi words, calls for an explanation, since it should be present in both the standards and deviants during the calculation of identity difference waves, and thus subtracted away. The removal of the SR-UR Mapping Positivity after subtraction indeed should be the case, under idealized circumstances; however, we posit that due to a habituation effect among the standards (Sams et al., 1984; Budd et al., 1998; Yagcioglu and Ungan, 2008), where extensive repetitions are present, the SR-UR Mapping Positivity should be weaker in the standards than in the deviants, resulting in residual SR-UR Mapping Positivity in the identity difference waves #2 and #4.

We acknowledge that our modeling regarding how the summation of three operations (SR-UR Mapping Positivity, UR-mismatch Positivity, and Base Positivity) could have led to the differential latency and amplitude profiles in the S1-S2 transitional region is highly speculative. Future research is necessary to verify or disconfirm our speculation.

##### Processing in S2

Figure 5 plots the identity difference wave of all conditions across the two syllables over selected electrodes. According to Figure 5, there is still processing going on in S2, despite all S2s being linguistically identical and acoustically homogeneous. If the processing of S2 is restricted to relaying acoustic signals, the identity difference waves in S2 should be flat. Importantly, in all conditions, the waveforms at the S1-S2 transitional region are first positive-going, then negative-going. This polarity reversal guarantees that the S2 negativity indeed reflects S2 processing, rather than a spill-over from an earlier time. Similar to the “Base Positivity” we hypothesized for the positivity at the S1-S2 transitional region in the above section, the origin and the function of the S2 negativity are unclear, waiting for future studies.

The findings in the S2 negativity are similar to that of the S1-S2 positivity in three aspects: (1) the Sandhi deviant conditions had earlier latency than the Non-sandhi deviant conditions; (2) the UR-mismatch conditions are less negative (thus, more positive) than the UR-match conditions; (3) the effect grew stronger in the second half of the experiment (see the Supplementary Material for details), suggesting the role of developing expectations as experiment processes.

Although all S2s are /ma^3^/ and have identical F0 contours, they may still differ in their recognition and disambiguation points, leading to different latency outcomes. Setting aside the possibility of an acoustically driven difference for the moment, the critical questions for understanding S2 processing are: (1) what kind of processing is going on in S2? (2) what does the S2 negativity index?

We could propose a mechanism similar to the one we offered for the S1-S2 transitional positivity. Under such a scenario, the brain keeps mapping SR to UR in S2, showing a UR-mismatch effect, similar to what has occurred in the S1-S2 transitional region. Nevertheless, it is unlikely that the same mechanisms would persist unaltered in S2. A different but related proposal would be as follows: the S2 negativity indicates a confirmation of the SR-UR mapping decision and the *UR Relation* decision attempted in the S1-S2 transitional region. Under such a scenario, S2 serves another function: the confirmation of the S1 mapping that was attempted. Under such a scenario, in the S1-S2 transitional region, the brain may be entertaining the possibility of mapping the SR to an underlying T3, depending on the information collected; in S2, which decisively signals UR of S1, the brain would then commit to one parsing decision, which may manifest as an earlier latency for Sandhi (T3 + T3) deviants.

In summary, we suggest three explanations for the findings regarding the S2 negativity: (1) a difference in recognition point and/or disambiguation point in S2; (2) a continuation of the SR-UR mapping effect and the UR-mismatch effect, already present in the S1-S2 transitional region; (3) a reflection of a confirmatory mechanism, which takes in the S2 information to map S1 to its correct UR decisively. More research, especially research using multisyllabic stimuli, preferably in another language, is needed to determine whether the S2 negativity is purely physically driven or contains clues to the cognitive operations involved.

## Conclusion

We accumulated three pieces of evidence that strongly suggest the presence of an SR-UR mapping process during the perception of an alternated sound involving neutralization (i.e., Mandarin T3 sandhi form): (1) the disappearance of MMN despite the apparent segmental conflict in S1; (2) an effect of *Deviant Sandhi Status*, which distinguishes T2 + T3 and T3 + T3 deviants, in the S1-S2 transitional region and S2; (3) the UR-mismatch conditions yielded a greater frontal positivity in both the S1-S2 transitional region and S2. Because the evidence for the SR-UR mapping occurs before S2, which determines the UR of S1, our results indicate that the perception of alternated sounds is also incremental, similar to the perception of un-alternated sounds. We attempted to explain the latency and the amplitude differences in the S1-S2 transitional region with three hypothesized underlying processing mechanisms with distinct timing and amplitude profiles (SR-UR Mapping Positivity, UR-mismatch Positivity, and Base Positivity). Future research testing more lexical items and other languages, as well as using other experimental paradigms without extensive stimulus repetition, is needed to further test our proposed model. Finally, our study further demonstrated that MMN is an effective tool for studying phonological alternation by showing that the brain can access the UR of alternated sounds in a passive-listening MMN experiment.

## Data Availability Statement

The datasets, together with the analysis scripts, for this study are available on request to the corresponding author.

## Ethics Statement

The studies involving human participants were reviewed and approved by the Human Subjects Committee at the University of Kansas (STUDY00144397). The patients/participants provided their written informed consent to participate in this study.

## Author Contributions

YZ came up with the research idea, conducted the experiment, ran statistics, interpreted the results, wrote the manuscript, and revised the manuscript. JZ and RF discussed the research idea and methods with YZ, interpreted the results, and revised the manuscript. All authors contributed to the article and approved the submitted version.

## Funding

This study was supported by the National Science Foundation (#BCS-1826547).

## Conflict of Interest

The authors declare that the research was conducted in the absence of any commercial or financial relationships that could be construed as a potential conflict of interest.

## Publisher's Note

All claims expressed in this article are solely those of the authors and do not necessarily represent those of their affiliated organizations, or those of the publisher, the editors and the reviewers. Any product that may be evaluated in this article, or claim that may be made by its manufacturer, is not guaranteed or endorsed by the publisher.

## Footnotes

^1^Yuan and Chen (2014) is a corpus study that found a statistically significant difference between T2 and the T3 sandhi form in their acoustic realization. For example, the T3 sandhi form has a lower F0 than T2.

^2^An alternated morpheme exhibits different surface realizations depending on its context. Alternation can be viewed as a type of phonology-morphology interaction (Hayes, 2009). The Mandarin 3rd tone sandhi that we investigated in this study is one type of phonological alternation: it is neutralizing, conditioned by a syntagmatic context, and it is productive in non-words (Zhang and Lai, 2010; Zhang and Peng, 2013). Other types of phonological alternation are common as well. For more examples of phonological alternation, see Hayes (2009).

^3^Chang et al. (2019) also tested Taiwanese sandhi pairs using the MMN paradigm. The Taiwanese sandhi pairs Chang et al. (2019) examined differed from the Mandarin 3rd tone sandhi in one important aspect: unlike Mandarin 3rd tone sandhi, the Taiwanese tone sandhi pairs in Chang et al. (2019) are not 100% productive (for Mandarin 3rd tone sandhi productivity, see Zhang and Lai, 2010; Zhang and Peng, 2013). Therefore, we do not consider the Taiwanese sandhi MMN experiment directly relevant to our study.

^4^Numbers in superscript correspond to Mandarin lexical tone categories. When such numbers occur in phonological transcription (using forward slashes), they represent UR. When numbers occur in phonetic transcription (using square brackets), they represent the canonical SR for the corresponding lexical tone.

^5^As is typical for MMN studies, our stimuli include a limited number of lexical items. This is due to the fact that each lexical item must be repeated many times in order to ensure a sufficient number of standard and deviant tokens for statistical analysis.

^6^For example, if a measured component is positive, only the positive area within the measurement window will be considered.

^7^Because the cluster-based permutation test only offers weak control of the family-wise error rate (FWER), the *tmax* permutation test, which offers strong control of the FWER, was also applied to evaluate the robustness of an effect whenever a significant cluster was found. All significant clusters reported in this paper were also significant when a follow-up *tmax* permutation test was conducted. Moreover, because relatively wide windows were used in cluster-based permutation tests, which could potentially miss more focal effects (Groppe et al., 2011a), *tmax* permutation tests using windows of 30 ms were also performed. For all *tmax* permutation tests, the data were first down-sampled to 200 Hz, as we did not expect to find effects shorter than 5 ms. No additional focal effect was found. In what follows, we only report results based on the initial cluster-based permutation tests.

^8^Although the deviants are phonologically identical in their onset (all have a /x/ onset), the acoustic realizations of these onsets are distinguishable due to influences from the following vowel(s). This is confirmed by the auditory impression of the experimenter and the acoustic examinations of the stimuli, consistent with previous studies (Jongman et al., 2000; McMurray and Jongman, 2011; Salverda et al., 2014).

^9^The term “recognition point” refers to the time at which an individual lexical item is recognized, and the term “disambiguation point” refers to the time at which the distinction between the standard and the deviant is recognized.

## Supplementary Material

Supplementary Figure 1 ERP responses to the standards and the deviants in all conditions. P300 latency is shaded in gray.

Supplementary Table 1 Duration information (measured in milliseconds) of the original stimuli: (1) the initial fricative /x/ without vocal-fold vibration; (2) the voicing portion of the first syllable (S1); (3) the voicing portion of the second syllable (S2); (4) the end of S2 characterized by strong creaky voice, not amenable to F0 manipulation. The last row is used for the duration normalization of the stimuli.

Supplementary Table 2 Intensity information (measured in dB) of the original stimuli: (1) the initial fricative /x/ without vocal-fold vibration; (2) the voicing portion of the first syllable (S1); (3) the voicing portion of the second syllable (S2); (4) the end of S2 characterized by strong creaky voice, not amenable to F0 manipulation. The last row is used for the intensity normalization of the stimuli.

Supplementary Table 3 A summary of the results of the split-half analysis.
