# Supplementary figures and images for: Electrophysiological Signatures of Perceiving Alternated Tone in Mandarin Chinese: Mismatch Negativity to Underlying Tone Conflict
Source: Front Psychol. 2021 Sep 27;12:735593. doi: 10.3389/fpsyg.2021.735593 (PMC8504678; doi:10.3389/fpsyg.2021.735593)

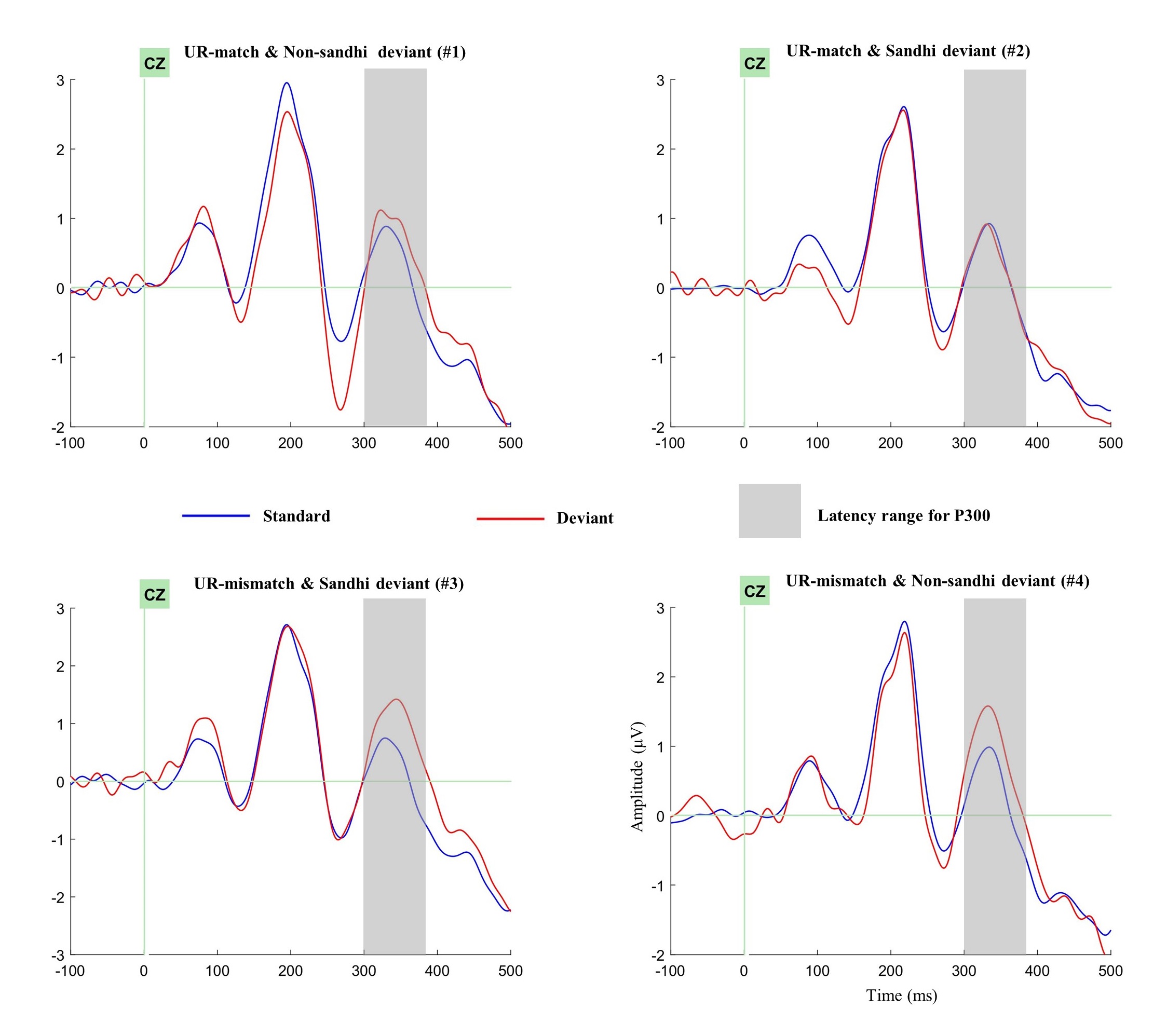

Supplement: Supplementary Figure 1 — ERP responses to the standards and the deviants in all conditions. P300 latency is shaded in gray. [file Image_1.JPEG]
