# Supplementary material for: Electrophysiological Signatures of Perceiving Alternated Tone in Mandarin Chinese: Mismatch Negativity to Underlying Tone Conflict
Source: Front Psychol. 2021 Sep 27;12:735593. doi: 10.3389/fpsyg.2021.735593 (PMC8504678; doi:10.3389/fpsyg.2021.735593)
Supplement: Supplementary Table 1 — Duration information (measured in milliseconds) of the original stimuli: (1) the initial fricative /x/ without vocal-fold vibration; (2) the voicing portion of the first syllable (S1); (3) the voicing portion of the second syllable (S2); (4) the end of S2 characterized by strong creaky voice, not amenable to F0 manipulation. The last row is used for the duration normalization of the stimuli. [file Presentation_2.pdf]

frontiers

Supplementary Table 1 Duration information (measured in milliseconds) of the original stimuli: (1) the initial fricative /x/ without vocal-fold vibration; (2) the voicing portion of the first syllable (S1); (3) the voicing portion of the second syllable (S2); (4) the end of S2 characterized by strong creaky voice, not amenable to F0 manipulation. The last row is used for the duration normalization of the stimuli.

**Stimulus**	**Portion**			
	**(1)**	**(2)**	**(3)**	**(4)**
**/xə^2 ma^3/**	185	282	176	190
**/xuaŋ^2 ma^3/**	187	231	287	199
**/xai^3 ma^3/**	176	241	247	290
**/xaʊ^3 ma^3/**	167	202	276	192
**Average**	179	239	247	218

Supplementary Table 2 Intensity information (measured in dB) of the original stimuli: (1) the initial fricative /x/ without vocal-fold vibration; (2) the voicing portion of the first syllable (S1); (3) the voicing portion of the second syllable (S2); (4) the end of S2 characterized by strong creaky voice, not amenable to F0 manipulation. The last row is used for the intensity normalization of the stimuli.

**Stimulus**	**Portion**			
	**(1)**	**(2)**	**(3)**	**(4)**
**/xə^2 ma^3/**	57.4	72.0	73.2	56.8
**/xuaŋ^2 ma^3/**	54.7	73.6	71.0	52.8
**/xai^3 ma^3/**	60.9	72.7	72.9	51.6
**/xaʊ^3 ma^3/**	60.3	72.4	72.3	50.4
**Average**	58.6	72.7	72.4	52.9

Supplementary Table 3 A summary of the results of the split-half analysis

**Effect**	**Window (ms)**	**Measure**	**Result**
MMN in the UR-match & Non-sandhi deviant Condition (#1)	200 to 300	Mean amplitude	First half > Second half ($p < 0.001$)
Early negativity in the UR-match & Sandhi deviant Condition (#2)	50 to 200		First half > Second half ($p < 0.001$)
S1-S2 transitional positivity (#1-#4)	250 to 400	Mean amplitude	First half < Second half ($p < 0.001$)
		Signed area amplitude	First half < Second half ($p < 0.001$)
S1-S2 transitional positivity as an effect of *UR relation* (#5 and #6)		Mean amplitude	First half < Second half ($p = 0.07$)
		Signed area amplitude	First half < Second half ($p = 0.08$)
S2 negativity (#1 - #4)	550 to 700	Mean amplitude	First half < Second half ($p < 0.001$)
		Signed area amplitude	First half < Second half ($p < 0.001$)
