# Supplementary material for: Electrophysiological Signatures of Perceiving Alternated Tone in Mandarin Chinese: Mismatch Negativity to Underlying Tone Conflict
Source: Front Psychol. 2021 Sep 27;12:735593. doi: 10.3389/fpsyg.2021.735593 (PMC8504678; doi:10.3389/fpsyg.2021.735593)
Supplement: Supplementary file 4 [file Presentation_1.pdf]

Split-half analyses

Split-half analyses were conducted by dividing the stimuli within each sub-block according to whether they occurred in the first/second half of a sub-block. The split-half analyses helped us evaluate: (1) the reliability of the reported effects; (2) whether a reported effect emerged due to participants developing expectations of the stimuli. In all split-half analyses, point measurements across the entire scalp were obtained. The results are summarized in Supplementary Table 3.

1 MMN and early negativity in S1

MMN in the UR-match & Non-sandhi deviant Condition (#1) is greater in the first half of the experiment ($p < 0.001$). Post-hoc tests revealed that while the first-half negativity was significantly negative, the second-half negativity was not (first half: mean amplitude = -0.67 μV, $p < 0.001$; second half: mean amplitude = -0.14 μV, $p = 0.70$). Stated differently, the MMN in the UR-match & Non-sandhi (#1) Condition was mainly present in the first half of the experiment: as participants habituated to our stimuli, MMN attenuated. Our finding of such an MMN habituation effect (i.e., MMN becoming smaller as the experiment continues) is consistent with previous reports (Dehaene-Lambertz & Dehaene, 1994; McGee et al., 2001; Rosburg et al., 2004; Woods & Elmasian, 1986).

The early negativity in the UR-match & Sandhi deviant Condition (#2) showed a similar habituation effect, with the early negativity being greater in the first half of the experiment ($p < 0.001$). However, in the mixed-effect modelling used for split-half analyses, neither was significantly negative (first half: mean amplitude = -0.13 μV, $p = 0.49$; second half: mean amplitude = 0.08 μV, $p = 0.94$).

In summary, both the MMN in the UR-match & Non-sandhi deviant Condition (#1) and the early negativity in the UR-match & Sandhi deviant Condition (#2) showed habituation. Participants show MMN from the beginning of the experiment, and this MMN gets smaller in the second half of the experiment. The observed MMN cannot be attributed to participants developing expectations as the experiment progresses, which would have been reflected in larger, rather than smaller, MMN as the experiment went on.

2 S1-S2 transitional positivity

Both measures of amplitude (mean amplitude and absolute positive area) obtained from the four identity difference waves that examined the behaviors of the deviants (#1 - #4) showed that the S1-S2 transitional positivity was stronger in the second half of the experiment (both *p*-values < 0.001), suggesting the development of expectations by the participants as the experiment progressed.

As for the two identity difference waves that directly evaluate the effect of *UR Relation* (#5 and #6), the second half was marginally more positive than the first half in the S1-S2 transitional region for both amplitude measures (all *p*-values < 0.08, two-tailed tests).

In brief, according to the split-half analysis, the S1-S2 transitional positivity reported as an effect of *UR Relation* grew stronger in the second half of each sub-block, suggesting that the participants developed expectations as the experiment progresses.

3 Negativity in S2

Both measures of amplitude (mean amplitude and absolute negative area) obtained from the four identity difference waves that examined the behaviors of the deviants (#1 - #4) showed that the S2 negativity was stronger in the second half of the experiment (both *p*-values < 0.001), suggesting the development of expectations by the participants as the experiment progressed.

References

Dehaene-Lambertz, G., & Dehaene, S. (1994). Speed and cerebral correlates of syllable discrimination in infants. *Nature*, *370*(6487), 292–295. https://doi.org/10.1038/370292a0

McGee, T. J., King, C., Tremblay, K., Nicol, T. G., Cunningham, J., & Kraus, N. (2001). Long-term habituation of the speech-elicited mismatch negativity. *Psychophysiology*, *38*(4), 653–658. https://doi.org/10.1111/1469-8986.3840653

Rosburg, T., Marinou, V., Haueisen, J., Smesny, S., & Sauer, H. (2004). Effects of Lorazepam on the neuromagnetic mismatch negativity (MMNm) and auditory evoked field component N100m. *Neuropsychopharmacology*, *29*(9), 1723–1733. https://doi.org/10.1038/sj.npp.1300477

Woods, D. L., & Elmasian, R. (1986). The habituation of event-related potentials to speech sounds and tones. *Electroencephalography and Clinical Neurophysiology/Evoked Potentials Section*, *65*(6), 447–459. https://doi.org/10.1016/0168-5597(86)90024-9
